# Context, design and conduct of the longitudinal COVID‐19 psychological research consortium study–wave 3

**DOI:** 10.1002/mpr.1880

**Published:** 2021-05-22

**Authors:** Orla McBride, Sarah Butter, Jamie Murphy, Mark Shevlin, Todd K. Hartman, Philip Hyland, Ryan McKay, Kate M. Bennett, Jilly Gibson‐Miller, Liat Levita, Liam Mason, Anton P. Martinez, Thomas VA Stocks, Frédérique Vallières, Thanos Karatzias, Carmen Valiente, Carmelo Vazquez, Richard P. Bentall

**Affiliations:** ^1^ Ulster University Coleraine Northern Ireland; ^2^ University of Sheffield Sheffield England; ^3^ Maynooth University Maynooth Republic of Ireland; ^4^ Royal Holloway University of London London England; ^5^ University of Liverpool Liverpool England; ^6^ University College London London England; ^7^ Trinity College Dublin Dublin Republic of Ireland; ^8^ Napier University Edinburgh Scotland; ^9^ Complutense University of Madrid Madrid Spain

**Keywords:** COVID‐19, general population, longitudinal, psychological, survey methodology

## Abstract

**Objectives:**

The COVID‐19 Psychological Research Consortium (C19PRC) Study aims to assess the impact of the COVID‐19 pandemic in the adult population in multiple countries. This paper describes the third wave of the UK survey (the ‘parent’ strand of the Consortium) during July‐August 2020.

**Methods:**

Adults (*N* = 2025) who participated in the baseline and/or first follow‐up surveys were reinvited to participate in this survey, which assessed: (1) COVID‐19 related knowledge, attitudes, and behaviours; (2) the occurrence of common mental disorders; as well as the role of (3) psychological factors and (4) social and political attitudes, in influencing the public’s response to the pandemic. Weights were calculated using a survey raking algorithm to ensure that the cross‐sectional sample is nationally representative in terms of gender, age, and household income, and representative of the baseline sample characteristics for household composition, ethnicity, urbanicity and born/raised in UK.

**Results:**

1166 adults (57.6% of baseline participants) provided full interviews at Wave 3. The raking procedure successfully re‐balanced the cross‐sectional sample to within 1% of population estimates across selected socio‐demographic characteristics.

**Conclusion:**

This paper demonstrates the strength of the C19PRC Study data to facilitate and stimulate interdisciplinary research addressing important public health questions relating to the COVID‐19 pandemic.

## 1 INTRODUCTION

Despite the existence of a substantial evidence base pointing to the positive sequelae of pandemics (e.g. increased resilience and optimism, better social support and bonding, etc.; Chen & Bonanno, 2020; Drury & Tekin Guven, 2020; Solnit, 2010), widespread concern has been expressed about the protracted nature of the COVID‐19 pandemic, and its potentially significant negative socio‐economic and health‐related impact on the lives of citizens over the medium to long term (Gayer˗Anderson et al., 2020; Ornell et al., 2020; Shah et al., 2020). By June 2020, over a quarter of a million people in the UK had contracted COVID‐19, and approximately 40,000 COVID‐19 related deaths had been registered (Office for National Statistics, 2020a). Approximately 8.9 million people were in receipt of income support via the government’s Coronavirus Job Retention Scheme (HM Revenue and Customs, 2020), and the UK debt level, which was estimated to be £1.95trn, was larger than the economy for the first time in over 50 years (Office for National Statistics, 2020b). Recent commentaries argue that the socio‐economic consequences of the pandemic are exposing and exacerbating existing societal inequalities, with the pandemic having a disproportionately negative impact on the lives of more vulnerable members of society (Morgan & Rose, 2020). Amidst these growing concerns, there is a pressing need to develop a robust evidence base, derived from analyses of high‐quality, population‐level data, to determine how the public are adapting to life and the many public‐health restrictions imposed throughout the course of the pandemic (Davis, 2020).

In March 2020, the longitudinal COVID‐19 Psychological Research Consortium (C19PRC) Study was designed and launched with the aim of collecting high‐quality data (via self‐report questionnaires, qualitative interviews, and quasi‐experimental studies) to test a range of theoretically‐informed research questions to obtain a greater understanding of the adult population’s psychological and social adjustments to the pandemic. Two core aspects of the C19PRC Study design will help ensure that this aim is achieved, and that the study’s data is well placed to contribute significantly to the knowledge base surrounding the mental health impacts of the COVID‐19 pandemic. First, a broad array of standardised measures were used to capture the prevalence of common mental disorders including major depressive disorder (MDD), generalized anxiety disorder (GAD), and posttraumatic stress disorder (PTSD), as well as other important experiences such as somatisation and paranoia (McBride et al., 2020). These core measures facilitate the assessment of a variety of mental disorders and experiences commonly investigated in previous infectious respiratory disease outbreaks (Cheng, 2004; Gardner & Moallef, 2015). They also offer a more detailed interrogation of these diagnostic constructs compared to other leading national longitudinal mental‐health studies currently being conducted during the pandemic, which have, in many cases, relied on established but general measures of psychological distress (Pierce, Hope, et al., 2020) or very short screening tools for MDD and GAD (Henderson et al., 2020).

Second, the inclusion of a battery of psychometric measures to assess individual‐level psychological factors (e.g., personality, memory, cognitive reasoning ability, locus of control, death anxiety, happiness, and resilience), political attitudes and behaviours (e.g., voting behaviour, political predispositions, nationalism, and patriotism), COVID‐19 health‐related knowledge and behaviours, as well as the collection of geo‐spatial data to facilitate linkage of individual‐level survey data to important macro‐level data (e.g., country‐level COVID‐19 related statistics including geographically‐framed infection rates, mortality rates, and lockdown status), ensures that the C19PRC Study possesses explanatory potential beyond that of most other studies and surveys established during the pandemic.

As detailed elsewhere, the C19PRC study commenced in the UK, but has since expanded to include international partners in the Republic of Ireland (RoI), Spain, Italy, Saudi Arabia, and the United Arab Emirates (UAE). The UK strand of the Study, to which we refer as C19PRC‐UK, is the ‘parent’ survey of the Consortium and is funded by the Economic and Social Research Council in the UK. Where possible/appropriate, international partners model their fieldwork procedures and survey content for each wave on the C19PRC‐UK design, although there are important differences between the countries in terms of the timing of fieldwork and survey content. For example, in the RoI and Spain, the first two waves were conducted during March​/April and May 2020 (Hyland et al., 2020; Valiente et al., 2020), which was consistent with the UK, whereas in Italy, the UAE and Saudi Arabia, baseline and follow‐up waves were conducted between April and August 2020 (Bruno et al., 2021). Whereas the UK survey has a strong focus on collecting socio‐political survey content (McBride et al., 2020), a key priority for the Spanish team was to measure and assess positive psychosocial responses to the pandemic (e.g., posttraumatic growth, hedonic and eudaimonic well‐being, openness to the future, primal positive beliefs, etc.; Valiente et al., 2020, 2021). The Consortium is committed to data harmonisation (where possible) to facilitate multi‐country research studies, and this complex programme of work is on‐going. Between April and September 2020, the Consortium produced 14 academic papers analysing the rich survey data, and several of these involved multi‐country data analysis (Hartman et al., 2020; Hyland et al., 2021; Murphy et al., 2021). All outputs are accessible via the dedicated OSF, COVID‐19 Psychological Research Consortium (C19PRC) Panel Study (2020) hosted with the Open Science Framework.

**FIGURE 1:**
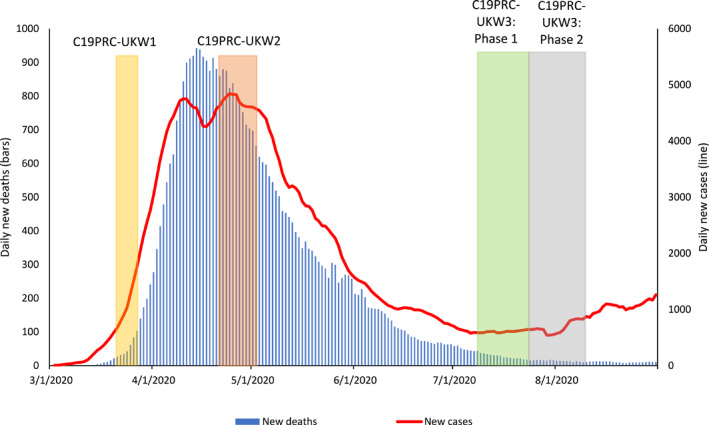
Graphical presentation of the number of daily COVID‐19 cases and deaths in the UK, sourced from Our World in Data, 2020, aligned to the C19PRC Study survey waves. New daily deaths and cases depicted as 7‐day rolling average

In this paper, we report the protocol for the third wave of C19PRC Study in the UK (C19PRC‐UKW3), which was conducted during July and August 2020. As described elsewhere (McBride et al., 2020), at baseline (C19PRC‐UKW1), 2025 adults aged ≥18 years, who were representative of the UK adult population with respect to gender, age, and household income, were recruited via an internet‐based panel survey in March 2020. Towards the end of April 2020, 1406 of these adults were recontacted for the first follow‐up survey (C19PRC‐UKW2), representing a 69.4% retention rate. The first two waves of the C19PRC Study were conducted at the beginning and peak of the first wave of COVID‐19 in the UK, respectively, whereas fieldwork for C19PRC‐UKW3 commenced at the tail end of the first wave (see Figure 1).

Despite the decline in daily COVID‐19 transmission and death rates, important social, economic, and political events rapidly unfolded during the period between the end of C19PRC‐UKW2 and C19PRC‐UKW3. These included, but were not limited to: (1) the relaxation of the first national lockdown; (2) commencement of human trials for a COVID‐19 vaccination in the UK; (3) social and political unrest during the pandemic; (4) the gradual return to school for children before the 2020 summer holidays; (5) announcement of a timeline to end the Coronavirus Job Retention Scheme, and (6) the introduction of travel‐related quarantine restrictions and bans (see Table S1 for a detailed timeline). As with previous waves, the content of the C19PRC‐UKW3 was considered carefully to capture the impact of these events on the lives of survey participants.

A key methodological concern of longitudinal panel studies is sample attrition (Lynn, 2009), and studies initiated during the COVID‐19 pandemic are not immune to this challenge. Attrition in a panel survey tends to increase as the number of follow‐up periods increases, and it has considerable potential to negatively impact on the generalisability of findings if participants who stay in the study differ from those who drop out in relation to core study outcomes (Gustavson et al., 2012). Whilst the C19PRC Study team works closely with our fieldwork partner, Qualtrics, to maximise the retention of adults across waves to protect and sustain the longitudinal credentials of the survey, refreshment or ‘top‐up’ sampling was conducted at C19PRC‐UKW3. Refreshment sampling recruits new respondents into the panel to match specific characteristics of adults who were lost to follow‐up. This process, which is common in established panel surveys such as the American National Election Study, ensures that the C19PRC panel sample will remain sufficiently large to conduct meaningful longitudinal analyses for the core study outcomes of common mental disorders, as well as being as representative as possible of the baseline target population (adults aged 18 years and older living in the UK). This paper describes the C19PRC team’s work to (i) examine the level of attrition in the C19PRC by the third wave and whether this could be predicted by important baseline mental‐health attributes, psychological characteristics, as well as socio‐demographic factors; (ii) conduct weighting procedures to formally manage attrition in the longitudinal panel; and (iii) determine the success of sample refreshment procedures conducted at C19PRC‐UKW3.

## 2 METHOD

### 2.1 C19PRC‐UKW3: Fieldwork procedures

#### 2.1.1 Fieldwork organisation overview

Fieldwork for the C19PRC Study was conducted by the survey company Qualtrics. Qualtrics partners with over 20 online sample providers to supply a network of diverse, quality respondents to their worldwide client base and, to date, has completed more than 15,000 projects across 2,500 universities worldwide.

#### 2.1.2 Procedure

C19PRC‐UKW3 survey data collection commenced on 9 July 2020, approximately 10 weeks after the completion of C19PRC‐UKW2. In Phase 1, Qualtrics re‐contacted all adults who participated in previous waves (*N* = 2025) via email, SMS, or in‐app notifications and invited them to participate. The survey was released to a sub‐sample of participants initially for a ‘soft launch’ (see **Quality Control Section**) prior to the full launch of the survey wave later that day.

Qualtrics’ partners released invitations in batches and, after the initial invitation was received, respondents who had not completed the survey were sent two reminders to encourage them to participate. The first reminder was sent approximately 36–48 h after the initial survey invite, with the second reminder sent another 36–48 h after this first reminder. Phase 1 fieldwork lasted two weeks (9–23 July 2020).

Prior to Phase 2, Qualtrics compared the characteristics of the Phase 1 sample to the pre‐determined sampling quotas set at baseline. As previously described (McBride et al., 2020), the target population for the C19PRC‐UKW1 survey was the UK adult population aged ≥18 years, and quota sampling methods were employed to achieve a representative sample in terms of age and gender (using 2016 population estimates from Eurostat, 2020) and household income (using 2017 income bands from the Office for National Statistics, 2017). Phase 2 fieldwork was therefore organised to recruit new respondents according to gaps in the sampling quotas following the completion of Phase 1. New respondents for Phase 2 were alerted to the C19PRC‐UKW3 by Qualtrics in one of two ways: (1) they opted to enter studies they were eligible for by signing up to a panel platform; or (2) they received automatic notification through a partner router which alerted/directed them to studies for which they were eligible. To avoid self‐selection bias, survey invitations to eligible participants only provide general information and do not include specific details about the contents of the survey. Participants were required to be adults, able to read and write in English, and resident in the UK. No other exclusion criteria were applied. Panel members routinely receive an incentive for survey participation (e.g., gift cards), based on the length of the survey, their specific panellist profile, and target acquisition difficulty.

Phase 2 fieldwork commenced on 23 July 2020 with a ‘soft launch’ (see **Quality Control Section**) and the full survey was launched on 24 July 2020. Qualtrics proceeded as follows during the Phase 2 fieldwork: (1) adults in ‘hard to reach’ quota groups (e.g., young people in the highest income bands) were targeted first; (2) the focus then shifted to allow the quotas to ‘fill up’ naturally; before (3) switching back to targeting respondents to fill incomplete quotas. Adults who chose to participate followed a link to a secure website and completed all surveys online. The invite link only remained active for a participant until a quota they would have qualified for was reached.

#### 2.1.3 Informed consent process

Participants were informed about the purpose of the C19PRC Study, that their data would be treated in confidence, that geolocating would be used to determine the area in which they lived (in conjunction with their residential postcode stem), and of their right to terminate participation at any time. Participants were also informed that some topics may be sensitive or distressing. Information about how their data would be stored and analysed by the research team was also provided. Participants were also informed that they would be re‐contacted at a later date to invite them to participate in subsequent survey waves. Participants provided informed electronic consent prior to completing the survey and were directed to contact the NHS 111 helpline upon completion if they had any concerns about COVID‐19.

#### 2.1.4 Compliance with general data protection regulation (GDPR)

C19PRC data will be stored confidentially in line with GDPR. When the study data is deposited with the UK Data Service, location data will be removed and replaced with relevant socioeconomic summary data (e.g. area‐level deprivation and population density data). All other personal data will also be removed.

#### 2.1.5 Quality control

Qualtrics conducted validation checks on the C19PRC‐UKW3 data, though this varied slightly across the Phases. In Phase 1, the ‘soft launch’ was conducted with 100 respondents and this data was screened for technical errors and omissions in the survey measures and/or filtering processes prior to the full launch. Adults who participated in the ‘soft launch’ were retained in the Phase 1 sample.

Qualtrics routinely analyses survey completion times to ensure that respondents spend sufficient time providing high‐quality responses. For longitudinal surveys, this process is completed once only, at baseline. Once a participant satisfies the minimum survey completion time, which is set at half the median time of the soft launch for that wave (11 min 11 s for C19PRC‐UKW1; McBride et al., 2020), the data they provide in subsequent waves is not subject to a minimum completion time restriction. Thus, the respondent’s completion time at baseline serves as an indicator of their status as a legitimate survey respondent which they carry with them across subsequent waves.

For Phase 2, Qualtrics screened the ‘soft launch’ data (*n* = 47) for technical errors and/omissions before the full launch and a survey completion time was again set based on half the median time for the soft launch (9 min, 42 s). Phase 2 ‘soft launch’ respondents were included in the main Phase 2 sample. Following the completion of Phase 2 fieldwork, Qualtrics removed any participants who (1) completed the survey in less than the minimum completion time or (2) were potentially duplicate respondents.

### 2.2 Measures

Table 1 provides an overview of the C19PRC‐UKW3 survey content by Phase (see Supplementary Materials for specific details of all measures administered).

**TABLE 1:** Overview of content^a^ of C19PRC Study Wave 3 (Phases 1 & 2), United Kingdom (UK), July–August 2020

Theme	Content	C19PRC wave 3	
		Phase 1	Phase 2
Demographics	Age, gender, country of residence, marital status, economic activity, key/essential worker status, born in the UK^†^, grow up in the UK^†^, urbanicity^†^, level of education^†^, religion^†^	X	X^†^only
Housing characteristics	Living alone	X	X
	Number of adults living in household	X	X
	Number of children living in household	X	X
	Ages of children living in household	X	X
	Housing tenure	‐	X
	Residential details (type of property; number of bedrooms; length at property)	X	X
Household finances	Estimated annual gross household income	‐	X
	Change in monthly household income during pandemic	X	X
	Use of savings/increasing debt during pandemic	X	X
	Made saving due to pandemic	X	X
	Perceived future financial security	X	X
Working hours	Changes in working hours (self)	X	X
Health conditions	Existence of any major underlying health conditions–self	‐	X
	Existence of any major underlying health conditions–immediate family member	‐	X
	Currently pregnant–self (partner)	X	X
	Number of weeks pregnant, if applicable	X	X
	Currently pregnant–immediate family member	X	X
Children in household	Childcare for children in household during lockdown	X	X
	Use of childcare facilities/services	X	X
COVID‐19	Sourcing of information (newspapers, TV, radio, social media, Internet, etc.)	‐	X
	Level of trust in information source	‐	X
	Engaging in behaviour to reduce risk of contracting COVID‐19 (e.g., wearing face mask)	X	X
	Engagement with lockdown restrictions	X	‐
	Anxiety‐level relating to COVID‐19	X	X
	Perceived individual risk contracting COVID‐19 over next 6 months	X	X
	Experiences of self‐isolation	X	X
	Experience of being infected with COVID‐19 (including testing) ‐ self	X	X
	Experience of having COVID‐19 (feeling unwell, admitted to hospital)	X	X
	Knowing someone close (family member/friend) who has tested positive for COVID‐19	X	X
	Knowing someone close (family member/friend) who has tested died due to COVID‐19	X	X
	COVID‐19 vaccine acceptability (self)	X	X
	COVID‐19 vaccine acceptability (child)	X	X
	Preference for schools reopening	X	X
	Comfort engaging in activities (e.g. socialising, shopping, going to the gym etc.)	X	‐
	Preference for pace of easing lockdown restriction	X	‐
	Predicted course of the pandemic	X	X
	Living in a local lockdown area	X	‐
	Concern about second coronavirus wave	X	X
	Support/opposition for restrictions in case of second wave	X	‐
	Support/opposition for air bridges and quarantine	X	‐
	Contact tracing: Knowledge and willingness to engage	X	‐
	Perceived compliance with social distancing: Neighbourhood, country, UK	X	X
	Perceived compliance with health and safety guidance: Neighbourhood, country, UK	X	‐
	Going on holiday/travel abroad	X	X
Mental health	Depression: *Patient health questionnaire‐9 (*Kroenke et al., 2001)	X	X
	Anxiety: *Generalized anxiety disorder scale‐7* (Spitzer et al., 2006)	X	X
	Traumatic stress *international trauma questionnaire* (Cloitre et al., 2018)	X	X
	Paranoia: *Persecution and deservedness scale* (Melo et al., 2009)	‐	X
	Somatic symptoms: *Patient health questionnaire‐15* (Kroenke et al., 2002)	X	X
	Self‐harm, suicidal thoughts and suicide attempts	X	X
	Social anxiety: *Mini social phobia inventory (mini‐SPIN)* (Connor et al., 2001)	X	‐
	Autistic traits: *Autism spectrum quotient (AQ‐10)* (Allison et al., 2012)	X	X
Psychological factors	Personality: *Big‐fiveiinventory‐10 (*Rammstedt & John, 2007)	‐	X
	Loneliness: *Loneliness scale* (Hughes et al., 2004)	X	X
	Death anxiety: *Death anxiety inventory* (Tomás‐Sábado et al., 2005)	‐	X
	Locus of control: *Locus of control scale* (Sapp & Harrod, 1993)	‐	X
	Self‐esteem: *Single‐item self‐esteem scale* (Robins et al., 2001)	X	X
	Resilience: *Brief resilience scale* (Smith et al., 2008)	‐	X
	Attachment style: *Relationships questionnaire* (Bartholomew & Horowitz, 1991)	X	X
	Hopefulness: *Brief‐H‐positive scale* (Fraser et al., 2014)	X	X
	Happiness: *Subjective happiness scale* (Lyubomirsky & Lepper, 1999)	X	X
	Life satisfaction	X	X
	Aspects of life better/worse since pandemic	X	‐
	Social support: *Modified medical outcome social support survey (mMOS‐SS)* (Ganz et al., 2003)	X	X
Health‐related behaviours	Alcohol use: *AUDIT‐C (*Bush et al., 1998 *)*	X	X
	Height and weight	X	X
Socio‐political views/related behaviours	Voting behaviour last general election	X	X
	Political party identification	X	X
	Voting behaviour European referendum	‐	X
	Measure of ‘left‐wing’ or ‘right‐wing’ on social and economic issues	‐	X
	Satisfaction with how government/institutions handling pandemic	X	‐
	Child rearing views	‐	X
	Experiences of discrimination (pre & during pandemic): *Everyday Discrimination Scale (short‐form)* (Sternthal et al., 2011)	X	‐
	Future voting behaviour	X	X
Trust	Institutions	X	X

^a^ Refer to Supplementary Material for detailed information on all study measures.

^†^ Variables indicates with this symbol were only administered at Phase 2.

#### 2.2.1 Study variables

The following C19PRC‐UKW1 variables were used for attrition analyses for C19PRC‐UKW3: gender (females vs. males); age (18–24 years olds vs. 25–34 years, 35–44 years, 45–54 years, 55–64 years, and 65+ years groups); household income (≤£15,490 per annum vs. £15,491–£25,340, £25,341–£38,740, £38,741–£57,903, and ≥£57,931 bands); ethnicity (White vs. other); education (post‐secondary education vs. other); economic activity (employed vs. other); urbancity (living in city vs. suburb, town or rural location); household composition (living alone vs. other; children <18 years living in household vs. other); living in UK (born or raised before aged 16 years in UK vs. other); physical health (self‐reported chronic health condition vs. other); probable MDD diagnosis (score of ≥10 on the *Patient Health Questionnaire‐9* vs. other); probable GAD diagnosis (score of ≥10 on the *Generalised Anxiety Disorder‐7* vs. other); probable PTSD diagnosis (using the *International Trauma Questionnaire’s* diagnostic algorithm for PTSD caseness vs. other); mental health treatment (current or past treatment for mental health problems vs. other); loneliness (score of ≥6 on the *Loneliness Scal*e); somatisation (total score on the *Patient Health Questionnaire‐15*); neuroticism (total score on the neuroticism subscale of the *Big‐Five Inventory‐10*); resilience (total score on the *Brief Resilience Scale*); paranoia (total score on the *Persecution and Deservedness Scale*); death anxiety (total score on the *Death Anxiety Inventory*); intolerance of uncertainty (total score on the *Intolerance of Uncertainty Scale*); and COVID‐19 anxiety (total score on single item indicator).

### 2.3 Ethical approval

Ethical approval for the project was provided by the University of Sheffield (Reference number 033759).

### 2.4 Data analysis plan and weighting procedures

Data analyses were conducted in a number of stages. First, the re‐contact rate for Phase 1 was calculated, and responders and non‐responders were compared on a range of baseline socio‐demographic, mental health, and psychological characteristics, using chi‐square tests and independent samples *t*‐tests. Second, a binary logistic regression analysis was conducted to assess the association between baseline characteristics and attrition at C19PRC‐UKW3. Regression coefficients (odds ratios and 95% confidence intervals) were plotted using the *coefplot* in Stata 15 (Jann, 2017; StataCorp., 2017).

Third, post‐stratification survey weighting was conducted for the Phase 1 sample using a technique known as survey raking or sample‐balancing, using the ‘anesrake’ package in *R* (Pasek & Pasek, 2018). Raking is a common method of adjusting survey data to ensure that the distribution of the characteristics of a sample closely mirror the known population distribution. In practice, this means the C19PRC‐UKW1 sampling quotas for age, gender, and household income, as well as the baseline proportions achieved for ethnicity, urbanicity, household composition, and being born or raised in the UK, were imposed on the sample obtained at Phase 1. The raking algorithm assessed which of these selected sociodemographic variable distributions at C19PRC‐UKW3 deviated from their target distribution at C19PRC‐UKW1 by 5% or more, and subsequently iteratively adjusted to produce a weight value for each case in the sample until the sample distribution aligned with the population distribution for the chosen characteristics (DeBell & Krosnick, 2009; Pasek & Pasek, 2018). Raking is considered an ideal method for weighting survey data given that it is relatively easy to implement, but also since it only requires the marginal population proportion for each variable used in the weighting procedure (Mercer et al., 2018). Weighted frequencies were calculated for baseline characteristics for C19PRC‐UKW3 Phase 1 sample to assess the success of the raking procedure.

And fourth, the representativeness of the combined C19PRC‐UKW3 Phase 1 and Phase 2 samples was assessed by comparing the characteristics of the sample to the UK general population. Standardised difference scores were computed using the *stddiffi* command in Stata 15 (Bayoumi, 2016; StataCorp., 2017) to test for differences in relation to specific socio‐demographic characteristics between the two data sources. Unlike other statistical tests (e.g. chi‐square), the standardised difference score approach is not influence by sample size (Austin, 2009), and can be more informative than *p*‐values for comparing across data sources that differ in relation to sample size (Harron et al., 2017). Standardised differences of 0.2, 0.5, and 0.8 represent small, medium, and large standardised differences respectively (Cohen, 1988); standardised difference scores of less than 0.1 suggests no meaningful differences between data sources in relation to the distribution of the variable under consideration (Normand et al., 2001).

## 3 RESULTS

### 3.1 Retention of respondents from previous waves

**FIGURE 2:**
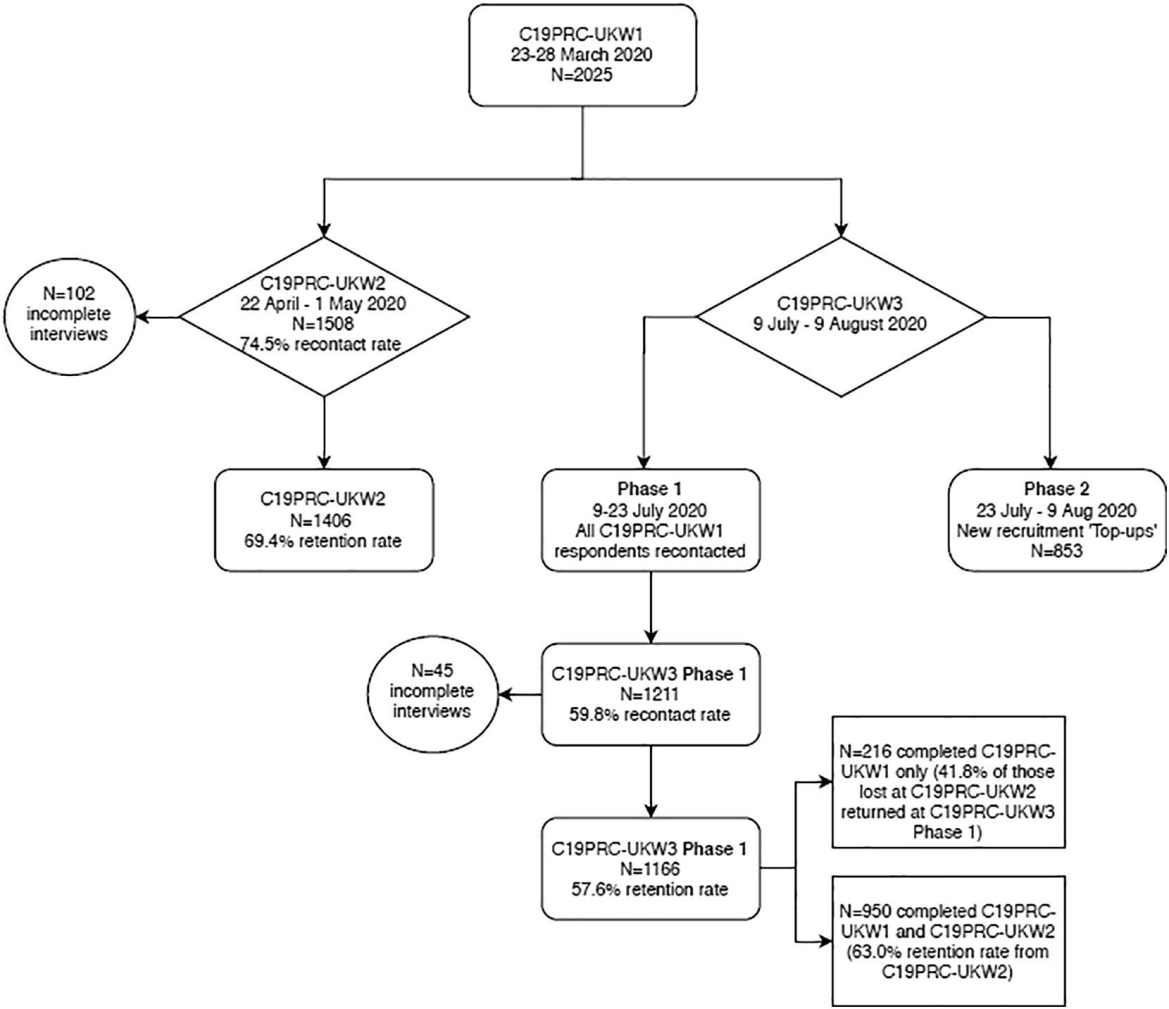
Flowchart of participation in the COVID‐19 Psychological Research Consortium Study (C19PRC) Study, Waves 1–3

As illustrated in Figure 2, at Phase 1, 1211 adults who participated in one or both of the previous waves were successfully recontacted (59.8% recontact rate) and 1166 adults provided full interviews at C19PRC‐UKW3 (i.e., 57.6% of baseline participants). This sample comprised 216 adults who completed C19PRC‐UKW1 only, meaning that 41.8% of adults who did not complete C19PRC‐UKW2 re‐entered the survey at C19PRC‐UKW3. The remainder of the Phase 1 sample comprised 950 respondents who had completed both previous survey waves, representing a 63.0% retention rate from C19PRC‐UKW2.

Table 2 compares the socio‐demographic and mental‐health related characteristics of survey respondents who participated in C19PRC‐UKW3 (*n* = 1166) compared to those who were lost‐to follow‐up (*n* = 859). In terms of socio‐demographic characteristics, higher proportions of adults lost to follow‐up were female, younger in age, had lower household incomes, were employed, were non‐White, were born outside the UK, lived in a city, had less than a post‐secondary level education, lived in a household with other adults, and had one or more children under 18 years living in their household. In terms of mental‐health characteristics and psychological factors, more adults lost to follow‐up had current or past experience of receiving treatment for mental health problems, met the caseness for depression, anxiety, and PTSD, were lonely, and had higher mean levels of neuroticism, somatisation, paranoia, death anxiety, intolerance of uncertainty, and lower mean levels of resilience.

**TABLE 2:** Attrition analysis for wave 3 of the COVID‐19 psychological research consortium (C19PRC) study (July–August 2020)

Wave 1 (baseline) respondent characteristics (March 2020)		Wave 1 respondents present at Wave 3 (*N* = 1166) N (%)	Wave 1 respondents absent at Wave 3 (*N* = 859) N (%)	*Test statistic* ^a^ ^,^ ^b^
Gender^a^	Male	620 (53.2%)	352 (41.0%)	30.014 (2), <0.001
	Female	542 (46.5%)	505 (58.8%)	
	Other	4 (0.4%)	2 (0.2%)	
Age group (years)^a^	18–24 years	68 (5.8%)	178 (20.7%)	215.484 (5) <0.001
	25–34 years	171 (14.7%)	209 (24.3%)	
	35–44 years	180 (15.4%)	173 (20.1%)	
	45–54 years	267 (22.9%)	143 (16.6%)	
	55–64 years	256 (22.0%)	93 (10.8%)	
	65+ years	224 (19.2%)	63 (7.3%)	
2019 household income^a^	≤£15.490	224 (19.2%)	186 (21.7%)	18.097 (4) 0.001
	£15,491–£25,340	216 (18.5%)	194 (22.6%)	
	£25,341–£38,740	212 (18.2%)	173 (20.1%)	
	£38,741–£57,903	245 (21.0%)	165 (19.2%)	
	≥£57,931	269 (23.1%)	141 (16.4%)	
Economic activity^a^	Employed (full or part‐time)	722 (61.9%)	569 (66.2%)	3.992 (1) 0.046
	Other	444 (38.1%)	290 (33.8%)	
Ethnicity^a^	White	1088 (93.3%)	760 (88.5%)	14.498 (1) <0.001
	Other	78 (6.7%)	99 (11.5%)	
Birthplace^a^	Born in UK	1078 (92.5%)	756 (88.0%)	11.432 (1) 0.001
	Born elsewhere	88 (7.5%)	103 (12.0%)	
Place of residence^a^	Suburb/Town/Rural	923 (79.2%)	604 (70.3%)	20.867 (1) <0.001
	City	243 (20.8%)	255 (29.7%)	
Educational attainment^a^	Post‐secondary education	726 (62.3%)	490 (57.0%)	5.620 (1) 0.018
	Did not attend post‐secondary education	440 (37.7%)	369 (43.0%)	
Religion^a^	Any religion	732 (62.8%)	525 (61.1%)	0.580 (1) 0.446
	Atheist or agnostic	434 (37.9%)	334 (38.9%)	
Household characteristics^a^	Single adult household	254 (21.8%)	145 (16.9%)	7.518 (1) 0.006
	Other	912 (78.2%)	714 (83.1%)	
	Children under 18 years living in household	263 (22.6%)	329 (38.3%)	59.267 (1) <0.001
	Other	903 (77.4%)	530 (61.7%)	
Physical health^a^	Chronic health condition	169 (14.5%)	142 (16.5%)	1.579 (1) 0.209
	None	997 (85.5%)	717 (83.5%)	
Mental‐health and psychological characteristics	Currently receiving/history of receiving treatment for mental health problems^a^	318 (27.3%)	279 (32.5%)	6.450 (1) 0.011
	Other	848 (72.7%)	580 (67.5%)	
	Depression–PHQ‐9 caseness met^a^	206 (17.7%)	242 (28.2%)	31.681 (1) <0.001
	Not met	960 (82.3%)	617 (71.8%)	
	Anxiety–GAD‐7 caseness met^a^	215 (18.4%)	223 (26.0%)	16.506 (1) <0.001
	Not met	951 (81.6%)	636 (74.0%)	
	PTSD caseness met^a^	158 (13.6%)	182 (21.2%)	20.647 (1) <0.001
	Not met	1008 (86.4%)	677 (78.8%)	
	Loneliness caseness met^a^	377 (32.3%)	369 (43.0%)	23.994 (1) <0.001
	Not met	789 (67.7%)	490 (57.0%)	
	Neuroticism^b^ [mean (SD)]	5.46 (2.11)	6.02 (2.07)	−5.926 (1870.19) 0.005
	Resilience^b^ [mean (SD)]	20.17 (5.06)	18.90 (4.91)	5.486 (1879.239) <0.001
	Somatisation^b^ [mean (SD)]	3.23 (4.58)	4.88 (5.60)	−7.057 (1621.092) <0.001
	Paranoia^b^ [mean (SD)]	11.70 (4.80)	13.47 (5.03)	−8.007 (2023) <0.001
	Death anxiety^b^ [mean (SD)]	41.87 (14.77)	46.36 (14.68)	−6.780 (2023) <0.001
	Intolerance of uncertainty^b^ [mean (SD)]	34.44 (9.02)	36.01 (9.22)	−3.818 (2023) <0.001
	COVID‐19 anxiety^b^ [mean (SD)]	67.46 (24.62)	68.08 (24.57)	−0.564 (2023) 0.573

^a^ Chi‐square *(df)*, *p*.

^b^ Independent samples test statistic (*df*), p.

### 3.2 Regression analyses–baseline characteristics predicting attrition at C19PRC‐UKW3

**FIGURE 3:**
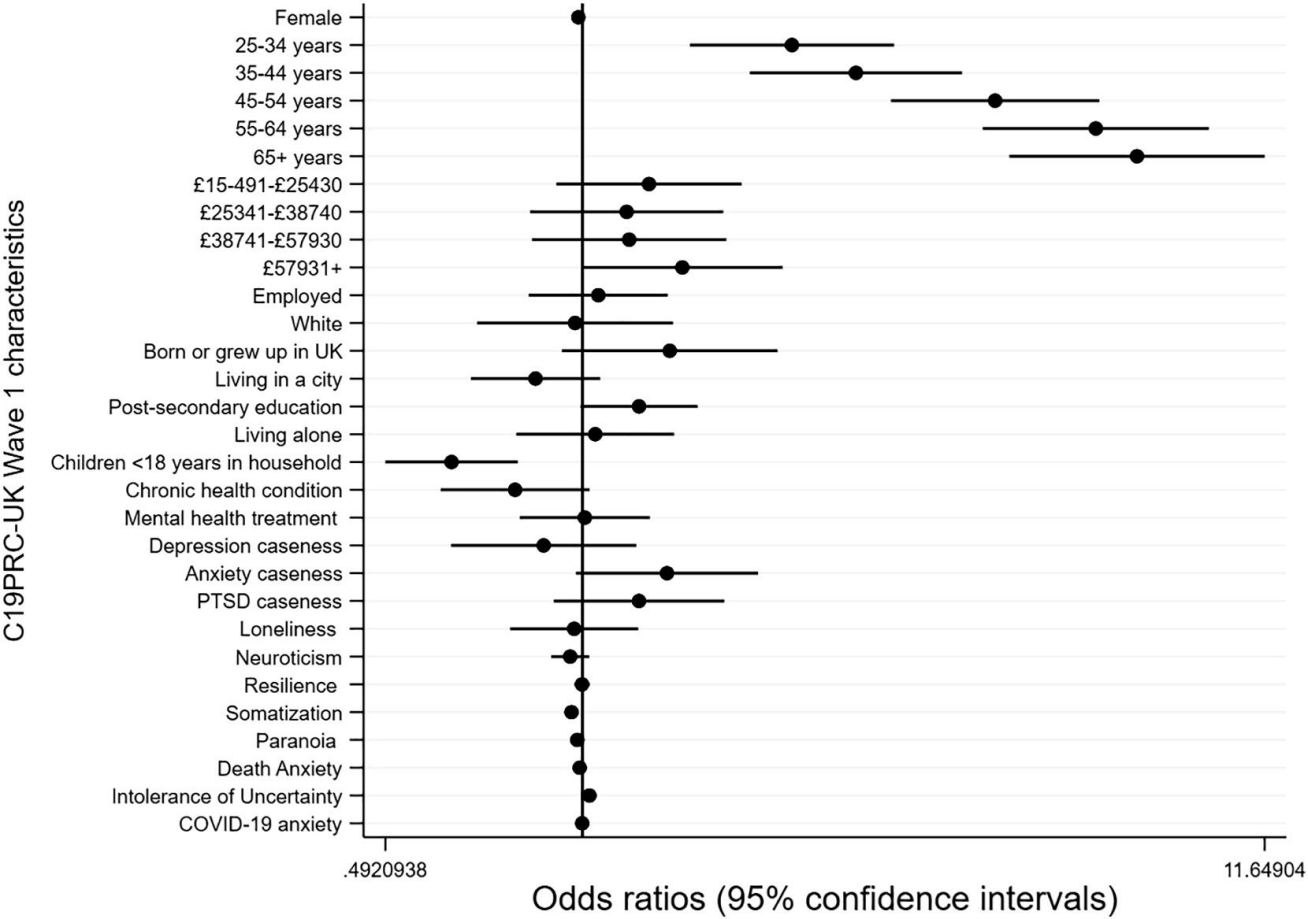
Plot of regression coefficients (and 95% confidence intervals) for baseline socio‐demographic, psychological and mental health characteristics (measured at C19PRC‐UKW1) predicting participation of adult respondents at the second follow‐up wave (C19PRC‐UKW3)

Figure 3 illustrates the results of the regression analyses estimating the associations between baseline characteristics and participation in the third wave (see Table S2 for model results). The vertical black bar represents an odds ratio of one, and the point estimates (odds ratios; OR) for each baseline characteristic are presented along with 95% confidence intervals (CI), which are indicated by horizontal black bars. Those which cross the vertical axis reflect a non‐statistically significant association between the baseline characteristic and attrition. Small to large effect sizes emerged for the association between age and attrition, with older adults experiencing greater odds of participating in the third wave compared to the 18–24 year olds as follows: 25–34 years (OR = 2.12; 95%CI 1.47–3.07); 35–44 years (OR = 2.67; 95%CI 1.82–3.92); 45–54 years (OR = 4.41; 95%CI 3.04–6.42); 55–64 years (OR = 4.22–9.53); and 65 years and over (OR = 7.35; 4.64–11.65). Adults with children under 18 years living at home had lower odds of participating in the third wave (OR = 0.62; 95%CI 0.49–0.79) compared to adults without dependants. Very small, but statistically significant associations emerged between higher levels of intolerance of uncertainty (OR = 1.03; 95%CI 1.01–1.04) and lower levels of somatisation (OR = 0.96, 95%CI 0.94–0.98) and non‐response at this wave; no other baseline mental‐health related characteristics, including caseness for depression, anxiety, or PTSD, were statistically significant predictors of attrition in the fully adjusted model.

### 3.3 Outcome of survey raking procedure for C19PRC‐UKW3 phase 1 sample

The raking procedure successfully re‐balanced the Phase 1 sample to the C19PRC‐UKW1 proportions for gender, age, household income (exact re‐balance to original quotas), household compositionand urbanicity (exact re‐balance to baselineproportions), ethnicity (within 0.3%), and born or raised in the UK (within 1%; see Table S3). The impact on the weighting on the baseline prevalence of the three core mental disorders measured in the C19PRC Study, MDD, GAD, and PTSD, was also assessed. Applying the weight variable re‐balanced the prevalence for each disorder as follows: depression (C19PRC‐UKW1, 22.1% vs. C19PRC‐UKW3 20.9%), anxiety (C19PRC‐UKW1, 21.6% vs. C19PRC‐UKW3 21.2%), and PTSD (C19PRC‐UKW1, 16.8% vs. C19PRC‐UKW3 16.8%).

### 3.4 Sampling quota recruitment at phase 2

Following an analysis of the outcome of Phase 1 recruitment, Phase 2 sampling quotas to target females, younger adults, and lower income earners. Overall, this process was successful–combining the samples across Phase 1 and 2 produced a cross‐sectional sample which closely mirrored the characteristics of the baseline sample with respect to gender (to within 0.1–0.5%, more males), age (to within 0.1‐0.3%, more older adults), and household income (to within 0.2–2.6%, with more higher‐income earners; see Table 3).

**TABLE 3:** Outcome of quota sampling recruitment, COVID‐19 Psychological Research Consortium (C19PRC) Study UK Wave 3 (C19PRC‐UKW3), July 2020

Socio‐demographic characteristics used for quota sampling		Quotas C19PRC‐UKW1	C19PRC‐UKW3 Phase 1 (*N* = 1166)		C19PRC‐UKW3 Phase 2 (*N* = 853)		C19PRC‐UKW3 Sample (Phases 1 & 2) (*N* = 2019)		Percentage difference between quota target and quota obtained at C19PRC‐UKW3
		%	n	%	n	%	n	%	
Sex^a^	Men	49	620	53.2	371	43.5	991	49.1	+0.1
	Women	51	542	46.5	477	55.9	1019	50.5	−0.5
	Other	NA	4	0.4	5	0.6	9	0.4	NA
Age group (years)^a^	18–24	12	68	5.8	175	20.5	243	12.0	0.0
	25–34	19	171	14.7	213	25.0	384	19.0	0.0
	35–44	18	180	15.4	189	22.2	369	18.3	+0.3
	45–54	20	267	22.9	138	16.2	405	20.1	+0.1
	55–64	17	256	22.0	85	10.0	341	16.9	−0.1
	65+	14	224	19.2	53	6.2	277	13.7	−0.3
Gross annual household income^b^	£0–£15,490	20	224	19.2	176	20.6	400	19.8	−0.2
	£15,491–£25,340	20	216	18.5	136	15.9	352	17.4	−2.6
	£25,341–£38,740	20	212	18.2	188	22.0	400	19.8	−0.2
	£38,741–£57,930	20	245	21.0	193	22.6	438	21.7	+1.3
	£57,931+	20	269	23.1	160	18.8	429	21.3	+1.3

^a^ Quotas for age and sex were derived from EUROSTAT 2016 population estimates (Eurostat, 2020).

^b^ Quotas for gross household income bands were on 2016 Office for National Statistics data (Office for National Statistics, 2017).

### 3.5 Representativeness of cross‐sectional C19PRC‐UKW3 sample

As presented in Table 4, the standardised differences scores resulting from comparisons of the C19PRC‐UKW3 sample to the UK population were all less than 0.1; this indicates that there were no meaningful differences in the distribution of the C19PRC‐UKW3 sample in terms of country of residence, born in the UK, and single adult household composition, compared to the UK‐wide and within country national adult populations.

**TABLE 4:** Comparison of representativeness of the COVID‐19 psychological research consortium (C19PRC) Study UK wave 3 (C19PRC‐UKW3) cross‐sectional sample to UK adult population for key socio‐demographic characteristics, by country, July–August 2020 (*N* = 2019)

		C19PRC‐UKW3 cross‐section (Phases 1 and 2 combined) *n* = 2019		Population		(+/− % difference between survey sample and population)	Standardised difference score
		n	%	n	%		
Between‐country composition							
Country of residence^a^	England/Wales	1800	89.1	42,645,389	88.7	0.4%	0.00030
	Scotland	185	9.2	4,109,000	8.5	0.7%	0.00005
	Northern Ireland	34	1.7	1,329,919	2.8	−1.1%	0.00290
Within‐country composition							
Born in UK^b^	England/Wales	1615	89.7	32,824,268	84.5	5.2%	0.00292
	Scotland	170	91.8	3,476,500	92.8	1.0%	0.00092
	Northern Ireland	33	97.1	1,277,369	92.6	4.5%	0.02284
Single adult household^c^	England/Wales	416	23.1	6,837,670	25.6	−2.5%	0.00265
	Scotland	47	25.4	1,221,359	33.1	−7.7%	0.00017
	Northern Ireland	9	26.5	NA	‐	‐	NA

^a^ Source. 2011 Census population estimates for adults aged 18+ years for England/Wales and Scotland; adults aged 20+ years in Northern Ireland.

^b^ Source. 2011 Census population estimates for adults aged 25+ years for England/Wales and Scotland; adults aged 18+ years for Northern Ireland.

^c^ Source. 2011 Census population estimates for adults aged 25+ years for England/Wales and Scotland; Northern Ireland provides publicly available data on household composition for the household reference person only (*N* = 703,275), not for all adults aged 18+ years, and therefore a comparison to survey for household composition was not feasible.

## 4 DISCUSSION

In this paper, we have demonstrated that by carefully designing a comprehensive psychological and mental‐health focused survey, which also prioritises the collection of data relating to the rapidly evolving public health and socio‐political context of the pandemic, the recruitment and retention of a large, nationally representative sample is achievable. By the third wave of the C19PRC Study, we have: (1) retained approximately 60% of baseline participants; (2) determined that the main predictors of attrition were sociodemographic in nature, specifically age and household composition (younger adults and those with dependants were less likely to participate), but less influenced by psychological factors or experiences of mental health disorders; (3) weighting procedures were largely able to account for this attrition‐related bias at C19PRC‐UKW3; and (4) sample replacement procedures were useful in bolstering the national representativeness of the sample in line with baseline sampling quotas, as well as the power of the sample for future longitudinal analyses.

Despite these strengths, we are cognisant of recent debate which questions the methodological quality of the vast array of mental‐health focused COVID‐19 research studies, many of which were set up in haste after the onset of the pandemic in late 2019. Chief among these concerns include the use of non‐probability, opt‐in online survey panels, the lack of comparable pre‐pandemic baseline data, and a reliance on unvalidated mental health measures (Holman et al., 2020; Pierce, Hope, et al., 2020; Pierce, McManus, et al., 2020). To those who might challenge the usefulness of C19PRC data on these grounds, we would like to highlight the following points.

It is undeniable that existing and established cohort and panel studies were in an optimal position to re‐focus data collection efforts to administer ‘COVID‐19 specific’ waves to their participants during the pandemic. Many of these studies having been set‐up in a pre‐pandemic era have the distinct advantage of being carefully planned and designed over many months, or even years, and have rightly adopted probability‐based sampling techniques. Whilst it is true that probability sampling has the advantage of permitting unbiased population estimates, recent evidence emerging from these ‘COVID‐19 informed’ waves, administered to existing participants, indicates they are experiencing lower than normal response rates. For example, only 48.6% of respondents who participated in the most recent wave of the UK Household Longitudinal Study (UKHLS), Wave 9 (2017–18), participated in the first UKHLS COVID‐19 web‐survey conducted during April 2020 (Pierce, McManus, et al., 2020), which is considerably lower than the reported 85% of UKHLS respondents who participated in Wave 9, having completed Wave 8 during 2016–17 (KANTAR, 2019). Experiencing sampling selection bias on a wide scale can impact negatively on estimated obtained from analyses of this survey data.

While we do not contest the argument that ‘*epidemiological enquiry is of little value unless a random sample is obtained*’ (p.57) (Tyrer & Heyman, 2016), we argue against recent position statements which suggest that (i) turnover in these types of panels is high; (ii) those who are in difficult financial circumstances complete surveys for financial gain, and (iii) self‐selected commercial survey panels might be biased towards mentally unhealthy or unhappy individuals (Chauvenet et al., 2020). Findings produced from analysis of our survey data provides evidence against these factors having a major influence in our study data.

First, we have demonstrated our ability to retain 57.6% of our baseline sample over two follow‐up surveys conducted during a five‐month period. Although critics may argue that this is evidence that participation in the C19PRC panel is decreasing (∼70% retention between baseline and first follow‐up compared to ∼60% retention between first and second follow‐ups), and that these attrition metrics hover at the boundary of acceptability, we believe they are comparable to other existing or established panel studies with short intervals between waves. For example, the 2008–2009 American National Election Study (ANES) conducted monthly interviews during the 2008 election cycle and lost 36% of respondents in less than a year (Deng et al., 2013). Given the unprecedented nature of the pandemic, however, it is questionable what the benchmark for acceptable levels of participation in repeated waves for a panel survey focusing on mental health should be. Thus, we sought to compare our participation rates to existing panel studies (e.g., UKHLS) that have re‐purposed fieldwork activities to administer COVID‐19 surveys. This would seem reasonable given that evidence suggests that whilst participation in cross‐sectional surveys had fallen dramatic in recent years, participation of respondents in existing longitudinal panel surveys has remained stable (Schoeni et al., 2013). Unfortunately, respondent retention rates across repeated waves of the UKHLS COVID‐19 surveys do not appear to have been reported; however, the response rates for these monthly surveys were low, declining from 48.6% in April 2020 (Wave 1) to 38.7% in July 2020 (Wave 4; UKHLS, 2020). Against this backdrop, we believe we have provided strong evidence as to the robustness of the C19PRC Study as a legitimate cohort study conducted during the worst public health crisis in living memory. Furthermore, we are committed to working closely with our fieldwork partners to re‐engaging all survey respondents at each wave, not just those who participated in the most recent wave, and this will provide an opportunity to establish whether this is true ‘drop out’ from the panel, or merely temporary due to respondents experiencing difficulties associated with the pandemic (e.g., illness) at the time of fieldwork.

Second, we sampled respondents based on quintiles of household income to ensure the sample was not over‐represented by those in financial difficulty, and our analysis revealed that our baseline sample was slightly over‐represented by people who were economically active (full‐time) (McBride et al., 2020).

Third, although the C19PRC‐UKW1 prevalence estimates of GAD and MDD (21.6% and 22.1%, respectively; Shevlin et al., 2020) were higher than estimates emerging from other UK adult population studies conducted before the pandemic (Giebel et al., 2020; Stansfeld et al., 2016), they were only marginally so, which suggests that the sample we recruited was not particularly mentally unhealthy. It could be argued, however, that it is not meaningful to compare prevalence estimates for mental disorders obtained during the pandemic via an online panel survey to those obtained from a probability‐based sample pre‐pandemic because the differences in mode of administration are intertwined with potential increases in prevalence estimates for mental disorders as a result of the pandemic. We are aware of one study in the US which demonstrated the ability of an online panel survey, using quota sampling methods, to produced remarkably similar prevalence estimates for PTSD when compared to a survey using probability‐base sampling in the pre‐pandemic era (Cloitre et al., 2019), which provides further confidence in our study’s data.

Much has been made of the ability of existing cohort studies to provide robust comparative analysis of ‘pre‐pandemic’ data on a range of important health outcomes to data collected ‘post‐pandemic’ (Henderson et al., 2020; Pierce, Hope, et al., 2020). Although several existing cohorts have employed mixed modes of survey administration in recent years–for example, 52% of interviews for Wave 9 of UKHLS were completed via web‐based interviews, compared to 47% completed via face‐to‐face interviews and 1% via telephone (KANTAR, 2019) ‐ the very nature of the pandemic forced a shift to an entirely web‐based mode of survey administration for all existing studies. It is an empirical question as to how comparable, precisely, data collected via different modes of survey administration are, even if they are collected from the same participants. Although it is likely too early in the pandemic to fully appreciate the actual differences produced by switching mode of administration, Zhang et al. (2017) demonstrated that mode of survey administration matters; in particular, social desirability effects are lower for surveys completed online compared to those administered face‐to‐face. Previous methodological work conducted in Israel also indicates that prevalence estimates for mental disorders such as PTSD can be considerably lower when individuals participate in face‐to‐face interviews compared to completing self‐report measures (Hoffman et al., 2011). In terms of mental health‐related outcomes, lower social desirability effects may mean respondents are more willing to report problems with their mental health in an online survey completed ‘during/post pandemic’ compared to the face‐to‐face survey pre‐pandemic, even if the face‐to‐face survey comprised of a confidential self‐report task in the presence of an interviewer. This results in what appears to be an increase in the prevalence estimates of mental disorders, which may be potentially a measurement artefact.

Even acknowledging the apparent superiority of the study design which re‐purposes existing cohort/panel fieldwork for the collection of data during the pandemic, we feel compelled to highlight that the measures administered to assess mental health in these established surveys are not optimal. For example, the UKHLS used the General Health Questionnaire (GHQ‐12; D. Goldberg & Williams, 1988) only, and, despite this being a recognised ‘gold standard’ for measuring general psychological distress reflective of potential cases of generalised anxiety and major depression (D. P. Goldberg et al., 1997), this scale does not actually measure these diagnostic entities (Mann et al., 2011). A key strength of the C19PRC study is the use of standardised instruments to measure specific diagnoses (i.e., MDD, GAD and PTSD) in accordance with the DSM‐5 and ICD‐11.

As a Consortium, we are committed to describing, in explicit detail, the context and planning stages of our survey data collection at each wave, and will continue to do so for future waves planned under the current UKRI ESRC funding programme, which expires in November 2021. We are planning a specific unique over‐sampling strategy at Wave 4 (taking place in November 2020) to secure robust sample sizes in each of the four nations of the UK to facilitate meaningful between‐country analyses on a range of factors (e.g. nation‐specific differences in experiences of and approaches to managing the pandemic), which we anticipate will differentially impact on individuals’ mental health and wellbeing as the pandemic continues to unfold.

## CONFLICT OF INTEREST STATEMENT

All authors declare no conflict of interest.

## Supporting information

Supplementary Material

Supplementary Material

## Data Availability

The data that support the findings of this study are available from the corresponding author upon reasonable request.
