# Supplementary material for: Context, design and conduct of the longitudinal COVID‐19 psychological research consortium study–wave 3
Source: Int J Methods Psychiatr Res. 2021 May 22;30(3):e1880. doi: 10.1002/mpr.1880 (PMC8209941; doi:10.1002/mpr.1880)
Supplement: Supplementary file 1 — Supplementary Material [file MPR-30-e1880-s002.docx]

Context, design and conduct of the longitudinal COVID-19 Psychological Research Consortium (C19PRC) Study – Wave 3

**Supplementary Material**

Short title: C19PRC Study Wave 3 protocol

Orla McBride^1*^, Sarah Butter^2^, Jamie Murphy^1^, Mark Shevlin^1^, Todd K. Hartman^2^, Philip Hyland^3^, Ryan McKay^4,^ Kate M. Bennett^5^, Jilly Gibson-Miller^2^, Liat Levita^2^, Liam Mason^6^, Anton P. Martinez^2^, Thomas VA Stocks^2^, Frédérique Vallières^7^, Thanos Karatzias^8^, Carmen Valiente^9^, Carmelo Vazquez^9^, & Richard P. Bentall^2, 6^

^1^ Ulster University, Northern Ireland

^2^ University of Sheffield, England

^3^ Maynooth University, Republic of Ireland

^4^ Royal Holloway, University of London, England

^5^ University of Liverpool, England

^6^ University College London, England

^7^Trinity College Dublin, Republic of Ireland

^8^Napier University, Scotland

^9^Complutense University of Madrid

**Corresponding author:** Dr Orla McBride, School of Psychology, Ulster University, Cromore Road, Coleraine, BT52 1SA, Northern Ireland. Email: [o.mcbride@ulster.ac.uk](mailto:o.mcbride@ulster.ac.uk); Tel: 0044(0)2870123987.

**2. Methods**

**2.2 Measures – C19PRC-UKW3**

**2.2.1 Socio-demographic characteristics.** Respondents provided data on their: age, gender (male, female, other), gross annual household income (which were used for quota sampling - £0-£15,490, £15,491-£25,340, £25,341-£38,740, £38,741-£57,930 and £57,931+) (***Phase 2 only***), religion (Christian; Muslim; Jewish; Buddhist; Sikh; Atheist; Agnostic; or other-specify) (***Phase 2 only***); urbanicity of residence (city; suburb; town; or rural area) (***Phase 2 only***); highest level of education (no qualifications; O-level/GCSE or similar; A-level or similar; diploma; undergraduate degree; postgraduate degree; technical qualification; or Other-specify) (***Phase 2 only***); legal marital or same-sex status (never married and never registered same-sex civil partnership (Single); separated, but still legally married; divorced; widowed; married; in a registered same-sex civil partnership; separated but still legally in a same-sex civil partnership; or formally in a same-sex civil partnership which is now legally dissolved; surviving partner from a same-sex civil partnership); born in UK (yes/no) (***Phase 2 only***); raised in UK (before age of 16 years) (yes/no) (***Phase 2 only***); and country of residence (England, Wales, Scotland, or Northern Ireland).

**2.2.2 Economic activity***.*

Respondents were asked to indicate whether they were: employed full-time, employed part-time (regular hours); self-employed (full-time), self-employed (part-time), zero hours contract, other flexible work practice; been placed on the government ‘furlough’ scheme; unemployed (because of coronavirus); unemployed (not because of coronavirus); full-time student; or retired. Respondents were asked ‘*As a consequence of the COVID-19 pandemic, have you worked*: (1) fewer hours per week than before the pandemic; (2) about the same amount of time per week as before the pandemic; (3) more hours per week than before the pandemic; (4) not applicable.. All respondents who indicated that they were not unemployed (not because of coronavirus), full-time student, or retired, were also asked to consider a definition of a ‘key worker’ (i.e. people whose jobs are vital to public health and safety during the coronavirus lockdown) and to determine whether their occupation was covered by any of the following categories (UK Cabinet Office/Department of Education, 2020): (1) Health and social care worker (e.g. all NHS staff including administrative and cleaning staff, care home workers); (2) Education and child care (e.g. nursery care workers and teachers); (3) Food and other necessary goods (e.g. staff involved in production, processing, distribution, sale and delivery of goods); (4) Key public services (e.g. postal workers, those required to run the justice system, religious staff, those responsible for managing the deceased and journalists providing public service broadcasting); (5) Local and national government (e.g. staff in administrative roles essential to the effective delivery of the COVID-19 response or delivering essential public services including payment of benefits); (6) Utility workers (e.g. staff needed to keep oil, gas, electricity, water and sewerage operations running, staff in the civil nuclear, chemical and telecom communications sectors); (7) Public safety and national security (e.g. police and support staff, Ministry of Defence civilian staff and armed forces personnel, fire and rescue staff, and workers responsible for border security, prisons, and probation); or (8) Transport (e.g. staff keeping air, water, road and rail passenger and freight transport modes operating).

**2.2.3 Housing characteristics.**

*2.2.3.1 Household composition.* Respondents provided information as to whether they lived alone or, if they lived with other people, how many adults and/or children aged younger than 18 years, lived with them. Respondents were also asked about housing tenure (own outright; own with a mortgage; shared ownership; renting; living rent free; or other). Data was collected about the age of each child (from 0-17 years) living in the household, where applicable, starting with the youngest child.

*2.2.3.2 Physical properties of place of residence*. Respondents were asked to indicate: (1) the type of property in which they live (a flat, house or bungalow); (2) how many bedrooms are in the property (ranging from 0 bedrooms, a single-room dwelling, studio or flat to 5 or more); and (3) how long they have lived at the property (under 1 year, 1-2 years, 3-5 years, or over 5 years).

**2.2.4 Household finances.**

*2.2.4.1 Income changes during the pandemic.* Respondents were asked to “Please estimate the percentage change (either increase or decrease) in their monthly household income compared to the average monthly income before the COVID-19” with response recorded on a visual slider ranging from 100% Less to 100% More, centred (starting point) at No change. Respondents were also asked “Are you making savings because of the COVID-19 pandemic (i.e. do you have more money at the end of the month)? And “Are you using savings to help your household manage during the COVID-19 pandemic?”, both scored using (1) yes, (2) no, or (3) Don’t know. Respondents were also asked “Has your overall debt increased or decreased this month due to COVID-19?”, with responses scored on a 5-point Likert scale ranging from (1) increased a lot, to (5) decreased a lot; respondents were also able to indicate a response of ‘I do not have debt’. Finally, respondents were asked to consider “*Looking forwards, do you expect your financial security to* (1) get worse, (2) stay about the same, or (3) get better?”

**2.2.5 Health conditions***.* The UK government directed that living with a major underlying health conditions is a risk factor for experiencing more severe ill-health and even death upon contracting COVID-19. At ***Phase 2 only***, participants were asked whether they and members of their immediate family were living with any major underlying health conditions (e.g. lung conditions, heart disease, kidney disease, liver disease, conditions affecting the brain and nerves, diabetes, problems with your spleen, a weakened immune system) (Yes/No response); All respondents were asked whether they or their partner were pregnant at the time of the survey and, if so, how many weeks? Respondents were also asked whether any members of their immediate family were pregnant at the time of the survey.

**2.2.6 Children in household.**

*2.2.6.1 Childcare.* Where applicable, information was requested in relation to who is taking care of children in the household during the lockdown while childcare facilities and schools are closed/unavailable: (1) mother or main female carer; (2) father or main male carer; (3) both parents equally; or (4) other (specify). Respondents were also asked “*Are you using childcare at present (e.g. childminders and childminder agencies, nanny or home child-carers, day nurseries, private nursery schools, Local Authority maintained nursery schools, pre-schools and playgroups, out of school clubs, etc.)?”.* Response options included (1) yes; (2) no; (3) would like to but childcare is currently unavailable; (4) childcare is available but I am not comfortable using it at the moment; (5) childcare is available but I cannot afford to use it. All respondents, regardless of whether they had children, were asked their opinion on schools reopening, as follows: “*Which of the following options for schools in the UK do you prefer?*” (1) schools and child care facilities should remain closed to most students; (2) all school and child care facilities should be open to all children, with social distancing in place; or (3) all schools should open as normal, with no social distancing.

**2.2.7 COVID-19.** As this study was devised in mid-March 2020 at the beginning of the pandemic when COVID-19 was a new virus, no existing measure was available to assess the general population’s knowledge, attitudes, and behaviours (KAB) of the virus. In order to assess COVID-19 related KAB, measures developed for use in studies of other global pandemics, for example the 2003 SARS outbreak, the 2009 H1N1 flu pandemic and the 2013-16 Ebola virus pandemic, were consulted and assessed for suitability and adaptation, where possible. Reliable and trusted web sources in the UK (e.g. Public Health England, the National Health Service; NHS) and internationally (e.g. the Centre for Disease Control, the WHO) were also consulted for current, evidence-based knowledge and information relating to the clinical presentation and transmission of COVID-19. Details of the newly devised questions/measures are described below.

*2.2.7.1 Sourcing information about COVID-19 and level of trust in sources*. ***Phase 2 only***. Respondents were asked about (1) how much information about COVID-19 that they had obtained from a variety of sources, including newspapers, television, radio, internet websites, social media, their doctor, other health professionals, government agencies, and family or friends?; and (2) how much trust they had in the information they got from each source? Responses were scored on a 4-point Likert scale ranging from 1 ‘None/Not at all’ to 4 ‘A lot’.

*2.2.7.2 Confidence in UK response to COVID-19. Phase 1 only.* A single question was asked to assess respondents’ general satisfaction with how the UK Government has handled the COVID-19 pandemic (yes/no response). A series of statements were also presented to respondents to ascertain the level of satisfaction (on a 5-point Likert scale ranging from 1, very dissatisfied to 5, very satisfied) with the measures put in place by the UK Government to fight the COVID-10 (Coronavirus) pandemic in relation to: (1) health care; (2) employment; (3) education; (4) business and enterprise; (5) child care; (6) care homes; (7) black, Asian, and minority ethnic communities; (7) scientific research; (8) travel and tourism; (9) immigration; and (10) policing.

*2.2.7.3 Behaviour change to reduce individual risk of contracting COVID-19*. Respondents were asked to report on whether and how they have changed behaviours relating to their personal care and health recently to reduce their personal risk of being infected by COVID-19 during the pandemic. Statements were posed as follows: “To reduce your risk of being infected by COVID-19, in the past week have you…”: (1) Worn a face mask; (2) Washed your hands with soap and water more often; (3) Used hand sanitising gel if soap and water were not available; (4) Used disinfectants to wash surfaces in your home more frequently; (5) Covered your nose and mouth with a tissue or sleeve when coughing or sneezing; (6) Avoided touching your eyes or mouth; (7) Sanitized the handles of shopping trolleys or baskets before shopping; (8) Avoided sharing items with people such as utensils, dishes, drinks and towels; and (9) Avoided close contact greetings with people outside your family (e.g. shaking hands, hugging). Response categories were no, occasionally, or whenever possible.

*2.2.7.4 Perception of compliance with social distancing rules.* Respondents were asked to consider the proportion of the population (expressed as a % on a visual slider scale, centred at 50%) in their own neighbourhood; their country (England, Wales, Scotland, or Northern Ireland) and the UK as a whole who they believe follow (1) social distancing rules and (2) health and safety guidance.

*2.2.7.5 Views on current and future state of the COVID-19 crisis.*  Data was collected as to whether respondents believed that the worst of the COVID-19 crisis in the UK was (1) behind us, (2) happening now, and (3) ahead of us. Respondents were also asked “*As the UK re-opens after lockdown, how concerned are you about the possibility of a second wave of the virus (i.e. another rise in the number of COVID-19 infections and deaths and pressure on the NHS*?), with responses reported on a 5-point Likert scale ranging from (1) not at all concerned to (5) extremely concerned. At ***Phase 1 only***, respondents were also asked: “*In the event of a second wave of COVID-19, to what extent you would oppose/support:* (1) closure of non-essential businesses; (2) stay-at-home orders; (3) mandatory wearing of face masks in public places; (4) government aid to small business; (5) tightening of social distancing measures; (6) quarantine of up to 14 days; (7) closure of schools; and (8) local lockdowns, with responses recorded on a 5-point Likert scale ranging from (1) strongly oppose, to (5) strongly support. On the day C19PRC-W3 fieldwork began, a new travel policy of ‘air bridges’ or ‘travel corridors’ came into effect between the UK and 75 other countries, meaning a relaxation of the requirement for people who arrive in the UK to quarantine for14 days (see Supplementary Table 1). Respondents were asked to extent to which they agree (on a 5-point Likert scale ranging from 1, strongly disagree to 5, strongly agree) with this policy. Finally, respondents were asked the extent to which they support or oppose (5-point Likert scale ranging from 1, strongly oppose to 5, strongly support) allowing visitors to enter the UK without a 14-day quarantine from a list of 10 countries: Italy, Spain, Canada, Australia, United States, China, Thailand, Uganda, India, South Korea (Department for Transport, 2020).

*2.2.7.6 Perceived risk of contracting COVID-19.* Respondents estimated on a visual slider (ranging from 0% on the left-hand side to 100% on the right-hand side) their perceived percentage risk of contracting COVID-19 within the next month, within 2-3 months, and within 4-6 months of the survey.

*2.2.7.7 Anxiety relating to COVID-19.* Respondents’ degree of specific anxiety about the COVID-19 pandemic was assessed using a single visual slider scale, ranging from 0 ‘not at all anxious’ on the left-hand side to 100 ‘extremely anxious’ on the right-hand side (centred at 50).

*2.2.7.8 Experiences of self-isolation.* Respondents were presented with a definition of self-isolation (‘*self-isolation means that you have COVID-19 symptoms, or if someone you live with has symptoms, you must not leave your home for between 7-14 days’*) and were asked whether they had self-isolated during the COVID-19 pandemic (Yes/No response).

*2.2.7.9 Experiences of COVID-19 infection - self.* Respondents were asked “To the best of your knowledge, to date, have you been infected by COVID-19?” (I’m not sure/yes/no/ response categories). Three additional statements were presented to those responding ‘I’m not sure’ to clarify their response, as follows: “I have had symptoms but I do not think I have been infected with the COVID-19 virus”, “I have not had symptoms but I still think I have been infected with the COVID-19 virus”, or “I have had symptoms and I think I may have been infected with the COVID-19 virus however I have not been tested” (selection of one option only permitted).

For those indicating a ‘yes’ response, a series of additional questions were asked. First, two statements were presented to clarify their testing history: “I have had symptoms and I think I have been infected with the COVID-19 virus, however I have not been tested” or “Yes, I have been tested for COVID-19 and the test was positive”. Respondents were then asked to report (1) how unwell they felt on a visual slider scale ranging from 0 ‘Not at all unwell’ to 100 ‘Extremely unwell’, centred at 50; and (2) whether they were admitted to hospital (Yes/No response);

*2.2.7.10 Testing for COVID-19 – other.* Respondents were asked (Yes/No/Not applicable response) whether anyone else from their household had been diagnosed with COVID-19 (confirmed by a test) and whether anyone from their extended family had been diagnosed with COVID-19 (confirmed by a test).

*2.2.7.11 COVID-19-related deaths.* Respondents were asked whether anyone close to them had died because of COVID-19, with response options including ‘Yes’, ‘No’ and ‘Unsure – not certain death was caused by COVID-19’.

*2.2.7.12 COVID-19 vaccine.* At the time this survey was designed, no vaccine was available for COVID-19. Two statements were presented to respondents to ascertain their views about a potential vaccine, adapting items from existing measures designed to access vaccine acceptability for other diseases (see below). In anticipation of a vaccine being developed in the future, respondents were asked “*If a new vaccine were to be developed that could prevent COVID-19, would you accept it for: (1) yourself (*Yes, No, Maybe); and (2) your child/children (Yes, No, Maybe, Not applicable).

*2.2.7.13 Relaxing of lockdown restrictions.* ***Phase 1 only****.* Around the time of the C19PRC-UKW3 fieldwork, the UK was experiencing a gradual re-opening of society (see Supplementary Table 1 for detailed timeline). Respondents were asked their opinion about whether the government should speed up, maintain, or slow down the pace at which it is relaxing social distancing/self-isolation measures to allow a gradual return to normal activities? They were also asked to consider life before the pandemic, and to report “how comfortable do you now feel now feel when considering: (1) meeting with friends and family outside your household; (2) shopping in supermarkets; (3) shopping in other shops; (4) going/returning to your place or work; (5) going to bars and restaurants; (6) going to hair/beauty salons, barbers, spas; (7) going to large public gatherings such as sporting or music events; (8) going to gyms, with responses scored on a 4-point Likert scale ranging from 1, not at all comfortable to 4, completely comfortable.

*2.2.7.14 Localised lockdowns.* ***Phase 1 only****.* Approximately two weeks prior to the commencement of the C19PRC-UKW3 fieldwork, the first localised lockdown was announced in Leicester (see Supplementary Table 1). Respondents were provided with a definition and aim of a local lockdown (i.e. a partial or full re-introduction of measures to control the spread of the coronavirus in a specific locality… to control the spread of the coronavirus pandemic by containing it within a particular area and so avoid re-imposing social distancing restrictions across the whole of the country), and asked whether they were living in an area that is currently under local lockdown (yes/no response).

2.2.7.15 *COVID-19 ‘Track and Trace’ app.* ***Phase 1 only*.** At the time of C19PRC-UKW3 fieldwork, progress was being made across the UK to develop and launch country-specific smartphone apps to help stop the spread of the virus by alerting members of the public when they come in close contact with a positive case of COVID-19 (see Supplementary Table 1). The first app was launched in Northern Ireland (StopCOVID NI) (Health and Social Care Public Health Agency, 2020) launched on 31 July 2020, followed by Scotland (Protect Scotland) (NHS Scotland Test and Protect, 2020) on 10 September 2020, with England and Wales due to launch a similar app (NHS COVID-19 app) (NHS Test and Trace, 2020) on 24 September 2020. Respondents were asked whether they: (1) were aware of the government’s plans to use a smartphone app to conduct ‘contact tracing’ in the population (yes/no response); and (2) own and use a smartphone (yes/no response). Next, respondents were presented with a series of statements and asked to report how likely they would be (on a 5-point Likert scale from 1, very unlikely to 5, very likely) to: (1) download the app to your phone; (2) use the app to report COVID-19 symptoms; (3) self-isolate if the app suggests you should; and (4) provide details about those whom you have been in contact with.

**2.2.8 Mental health.** Experiences of mental health difficulties are core outcomes for the C19PRC Study. A key objective of the study was to administer a range of brief, standardised questionnaires to screen for the presence of common mental disorder, which would be repeated across all survey waves. Details are included below.

*2.2.8.1* *Patient Health Questionnaire-9 (PHQ-9*) (Kroenke, Spitzer, & Williams, 2001). Depression was assessed with the PHQ-9, a nine-item measure which corresponds to the DSM-IV Diagnostic Criterion A symptoms for major depressive disorder (American Psychiatric Association, 2000). Participants were asked how often, over the last two weeks, they had been bothered by each of the depressive symptoms. Response options were “not at all”, “several days”, “more than half the days”, and “nearly every day”, scored as 0, 1, 2 and 3, respectively. PHQ-9 scores range from 0 to 27, with scores of ≥5, ≥10, ≥15, representing mild, moderate and severe levels of depression severity (Kroenke et al., 2001). A threshold of ≥10 was used in this study. Psychometric properties of the PHQ-9 are well documented (see Kroenke, Spitzer, Williams, and Löwe (2010) for an overview).

*2.2.8.2 Suicidality.* Following the PHQ-9, the last item of which asks respondents about whether they experienced thoughts of dead or self-harm in the last two weeks, respondents were asked if they would be content to answer a series of questions relating to thoughts and actions of self-harm and suicide (adapted from the 2014 English Adult Psychiatric Morbidity Survey (McManus, Bebbington, Jenkins, & Brugha, 2016)). Respondents who answered ‘No’ were automatically skipped to the next measures (GAD-7, see next sub-section); respondents who answered ‘Yes’ were presented with the following statement to assess lifetime suicidal ideation: “*There may be times in everyone's life when they become very miserable and depressed and may feel like taking drastic action because of these feelings. Have you ever thought of harming yourself or taking your life, even if you would not really do it*?” (Yes/No response). Next, the respondent was asked to re-consider the statement but with specific reference to the time period since the pandemic began (Yes/No response). Respondents were then asked, “Have you ever made an attempt to take your own life?” (Yes/No response). Filtering was imposed depending on the respondent’s answer: (1) adults who responded ‘No’ were asked if they had ever deliberately harmed themselves in any way but not with the intention of taking your own life (Yes/No response) and, if yes, was this in (a) the last two weeks and/or (b) in the last year but not in the last two weeks (Yes/No response to both questions); (2) adults who responded ‘Yes’ were asked if the attempt to take their own life happened in (a) the last two weeks and/or (b) in the last year but not in the last two weeks (Yes/No response to both questions). At the end of the survey, all respondents were presented with contact information for self-harm or suicide support services, regardless as to whether they screened into this suicidality section or not.

*2.2.8.3 Generalized Anxiety Disorder Scale (GAD-7)* (Spitzer, Kroenke, Williams, & Löwe, 2006). Experiences of generalized anxiety were assessed using the GAD-7. Respondents were asked to report, on a 4-point Likert scale ranging from 1 (not at all) to 4 (nearly every day), how often in the past 7 days they were bothered by seven anxiety symptoms (e.g. trouble relaxing, becoming easily annoyed or irritable). The GAD-7 was originally validated in a primary care sample and a cut-off score of 10 had a sensitivity value of 0.89 and a specificity value of 0.82 for identifying generalised anxiety disorder (Spitzer et al., 2006), and a threshold of 10 was used in this study. The GAD-7 has demonstrated good reliability and construct validity, as evidenced by strong associations with other established measures of anxiety as well as diagnoses of GAD and its associations with depression, self-esteem, life satisfaction, and resilience (Löwe et al., 2008).

*2.2.8.4 International Trauma Questionnaire (ITQ)* (Cloitre et al., 2018). Post-traumatic stress disorder was assessed using the ITQ, a self-report measure of ICD-11 PTSD based on a total of six symptoms across the three symptom clusters of Re-experiencing, Avoidance, and Sense of Threat; each symptom cluster is comprised of 2 symptoms. Participants were asked to complete the ITQ as follows: “…in relation to your experience of the COVID-19 pandemic, please read each item carefully, then select one of the answers to indicate how much you have been bothered by that problem in the past month”. The PTSD symptoms are accompanied by three items measuring functional impairment caused by these symptoms. All items are answered on a 5-point Likert scale, ranging from 0 (Not at all) to 3 (Extremely) with possible PTSD scores ranging from 0 to 24. A score of ≥ 2 (Moderately) is considered ‘endorsement’ of that symptom. A PTSD diagnosis requires traumatic exposure, and at least one symptom to be endorsed from each PTSD symptom cluster (Re-experiencing, Avoidance, and Sense of Threat), and endorsement of at least one indicator of functional impairment. The psychometric properties of the ITQ scores have been demonstrated in multiple general population (Ben‐Ezra et al., 2018; Cloitre et al., 2019) and clinical and high-risk samples (Hyland et al., 2017; Karatzias et al., 2016; Vallières et al., 2018) samples.

*2.2.8.5 Patient Health Questionnaire-15 (PHQ-15)* (Kroenke, Spitzer, & Williams, 2002). The PHQ-15 is a brief, self-administered questionnaire which assesses for the presence and severity of the most prevalent DSM-IV somatization disorder somatic symptoms (American Psychiatric Association, 2000). Respondents rated the severity of symptoms, such as stomach pain, headaches, dizziness, they experienced over the last seven days as 0 (‘not bothered at all’), 1 (‘bothered a little’) or 2 (‘bothered a lot’). PHQ-15 score ranges from 0 to 30 and scores of ≥5, ≥10, ≥15 represent mild, moderate and severe levels of somatization (Kroenke et al., 2002). The reliability and validity of the PHQ-15 are high in clinical and occupational health care settings (de Vroege, Hoedeman, Nuyen, Sijtsma, & van der Feltz-Cornelis, 2012; Kroenke et al., 2002; Kroenke et al., 2010).

*2.2.8.6 Persecution and Deservedness Scale* (PaDS) (Melo, Corcoran, Shryane, & Bentall, 2009). ***Phase 2 only***. Paranoia was assessed with five items taken from the persecution subscale of the persecution and deservedness scale (PaDS), a measure designed for use with both clinical and population samples and which has been validated against both questionnaire and clinical measures of paranoia (Elahi, Algorta, Varese, McIntyre, & Bentall, 2017; Melo et al., 2009). Participants rated their agreement on a 5-point scale with statements such as “I’m often suspicious of other people’s intentions towards me” and “You should only trust yourself.” Response options ranged from 1 = strongly disagree to 5 = strongly agree. Scale reliability for the five items was very good (α = 0.84) in a previous epidemiological study of UK citizens (McIntyre, Wickham, Barr, & Bentall, 2018).

*2.2.8.7 Autism Spectrum Quotient (AQ-10)* (Allison, Auyeung, & Baron-Cohen, 2012). The 10-item AQ, an abbreviated version of the Autism Quotient (AQ) (Baron-Cohen et al., 2009), is a brief screening tool used by health professionals to determine whether children or adults require an in-depth clinical assessment for autism spectrum conditions. The AQ-10 contains the following items: 1 “I often notice small sounds when others do not”; 2 “I usually concentrate more on the whole picture, rather than the small details”, 3 “I find it easy to do more than one thing at once”, 4 “If there is an interruption, I can switch back to what I was doing very quickly”, 5 “I find it easy to ‘read between the lines’ when someone is talking to me”, 6 “I know how to tell if someone who is listening to me is getting bored”, 7 “When I’m reading a story, I think it difficult to work out the characters’ intentions”, 8 “I like to collection information about categories of things (e.g. types of car, types of bird, types of train, types of plant, etc.), 9 “I find it easy to work out what someone is thinking or feeling just by looking at their face”, and 10 “I find it difficult to work out people’s intentions”. A 4-point Likert scale is presented for each item (definitely agree to definitely disagree) and a score of 1 is assigned to responses of ‘definitely or slightly agree’ on items 1, 7, 8, and 10 and to responses of ‘definitely or slightly disagree’ on the other items. For adults, a cut-off of 6 has demonstrated sensitivity (0.88), specificity (0.91) and positive predictive value (0.85) compared to the full AQ for identifying individuals with autism spectrum conditions (Allison et al., 2012).

*2.2.8.8 Mini Social Phobia Inventory (Mini-SPIN)* (Connor, Kobak, Churchill, Katzelnick, & Davidson, 2001). *Phase 1 only.* The Mini-SPIN is a screening tool for generalised anxiety disorder. Derived from the 17-item Social Phobia Inventory (SPIN) for the assessment of generalised social anxiety disorder, the three-item screener tool comprises the following items (scored on a 5-point Likert scale ranging from 1, not at all to 5, extremely): ‘Fear of embarrassment causes me to avoid doing things or speak to people’, ‘I avoid activities in which I am the centre of attention’, and ‘Being embarrassed or looking stupid are among my worst fears’. Connor et al. (2001) demonstrated that, when compared to the Structured Clinical Interview (SCID) for DSM-IV generalised anxiety disorder, the Mini-SPIN (cut off score of >6) had good sensitivity (88.7%), specificity (90.0%), positive predictive value of 52.5% and negative predictive value of 98.5% for generalised anxiety disorder.

**2.2.9 Psychological factors.**

*2.2.9.1 Big-Five Inventory (BFI-10)* (Rammstedt & John, 2007). ***Phase 2 only.*** The five personality traits of openness to experience, conscientiousness, extroversion, agreeableness and neuroticism were assessed using the BFI-10, which contains items two items per personality construct such as ‘I see myself as someone who is reserved’, ‘ I see myself as someone who tends to be lazy’, and ‘I see myself as someone who has few artistic tendencies’. Rammstedt and John (2007) reported good reliability and validity for the 10-item scale.

*2.2.9.2 Loneliness Scale* (Hughes, Waite, Hawkley, & Cacioppo, 2004). Social connectedness was measured using the three-item Loneliness Scale, which was specifically designed for use in large-scaled population surveys (Hughes et al., 2004). Respondents were asked how often they felt: (1) that they lacked companionship; (2) left out; and (3) isolated from others. Responses were scored on a 3-point scale (hardly ever, sometimes, or often).

*2.2.9.3 Single-Item* *Self-esteem Scale* (SISES) (Robins, Hendin, & Trzesniewski, 2001). Respondents’ reported the extent to which they agreed with a single statement (‘I have high self-esteem’) on a 7-point Likert scale ranging from 1 ‘not very true of me’ to 7 ‘very true of me’. The SISES has been shown to have good convergent validity against other self-esteem measures (Robins et al., 2001).

*2.2.9.4 Hopefulness (Brief-H-Pos Scale)* (Fraser et al., 2014). Respondents were asked to complete the Brief-H-Pos Scale, a two-item measure which is a positive re-framing of the negatively worded two-item Hopeless Scale (Everson et al., 1996). The two items, “The future seems to me to be hopeful and I believe that things are changing for the better” and “I feel that it is possible to reach the goals I would like to strive for”, are scored on a 5-point Likert scale ranging from 1, absolutely disagree to 5, absolutely agree (higher scores indicate higher levels of hopefulness). Fraser et al. (2014) demonstrated that the scale had good internal consistency, test-re-test reliability and concurrent validity (compared to the Beck Hopelessness Scale (Beck, Weissman, Lester, & Trexler, 1974) and the Centre for Epidemiological Studies on Depression Scale (Radloff, 1977)), and recommended the scale as a useful screener for hopefulness in large general population studies.

*2.2.9.5 Subjective Happiness Scale (SHS)* (Lyubomirsky & Lepper, 1999). The SHS is a 4-item self-report measure of self-reported happiness. Respondents are to consider a series of statements (e.g. “Some people are generally very happy. They enjoy life regardless of what is going on, getting the most out of everything. To what extent does this characterization describe you?”) and report on a 7-point Likert scale (ranging from 1, Not at all to 7, A great deal). A single composite score is computed by averaging the responses to the four item (fourth item is reverse coded) and higher scores reflect higher levels of happiness. The SHS has high internal consistency, good to excellent reliability, and strong convergent and discriminant validity (Lyubomirsky & Lepper, 1999).

*2.2.9.6 Life satisfaction.* A series of questions were generated to assess respondents’ satisfaction with life during the pandemic. First, respondents were asked “Thinking about your life as it is right now, how satisfied are you with your life?” and presented with a visual slider indicating ‘level of satisfaction’ ranging from 0 ‘Completely unsatisfied’ to 100 ‘Completely satisfied’ centred at 50.

*2.2.9.7 Brief Resilience Scale (BRS)* (Smith et al., 2008). ***Phase 2 only.*** Respondents’ level of resilience was assessed using the 6-item BRS, which included the items such as: ‘I tend to bounce back quickly after hard times’; ‘ I have a hard time making it through stressful events’; and ‘ I tend to take a long time to get over set-backs in my life’. Items were scored on a 5-point Likert scale ranging from 1 ‘strongly disagree’ to 5 ‘strongly agree’, with items 2,4 and 6 reverse coded. The BRS has demonstrated construct, convergent, and discriminant validity in the general population (Kyriazos et al., 2018; Rodríguez-Rey, Alonso-Tapia, & Hernansaiz-Garrido, 2016).

*2.2.9.8 Locus of control (LoC) scale* (Sapp & Harrod, 1993). ***Phase 2 only.*** The short 9-item version of Levenson’s LoC scale (Levenson, 1973), which measures internal LoC (items such as ‘My life is determined by my own actions’) and external LoC, which has two components - change (items such as ‘To a great extent, my life is controlled by accidental happenings’) and powerful others (items such as ‘Getting what I want requires pleasing those people above me’). The internal, chance, and powerful others subscales were each measured by three questions using a 7-point Likert scale ranging from 1’ strongly disagree’ to 7 ‘strongly agree’.

*2.2.9.9* *Death Anxiety Inventory (DAI)* (Tomás-Sábado, Gómez-Benito, & Limonero, 2005). ***Phase 2 only***. Respondents’ attitudes towards death were assessed using the 17-item DAI, which measures four death-related anxiety factors (labelled as death acceptance, externally generated death anxiety, death finality, and thoughts about death) with items such as ‘I get upset when I am in a cemetery’, ‘The sight of a corpse deeply shocks me’, ‘I find it difficult to accept the idea that it all finishes with death’ and ‘I find it really difficult to accept that I have to die’. Responses were scored on a 5-point Likert scale ranging from 1 ‘totally disagree’ to 5 ‘totally agree’ (Tomás-Sábado et al., 2005).

*2.2.9.10 Relationships Questionnaire (RQ)* (Bartholomew & Horowitz, 1991). The RQ is a four-item scale designed to measure adult attachment styles. Respondents are presented with four statements capturing general relationship styles (A-D) and asked to indicate which letter best describes them, and then to indicate on a 7-point Likert scale, ranging from 1 ‘Strongly disagree’ to 7 ‘Strongly agree’, how well or poorly each description corresponds to your general relationship style: Style A (*It is easy for me to become emotionally close to others. I am comfortable depending on them and having them depend on me. I don’t worry about being alone or having others not accept me*); Style B (I am uncomfortable getting close to others. I want emotionally close relationships, but I find it difficult to trust others completely, or to depend on them. I worry that I will be hurt if I allow myself to become too close to others); Style C (*I want to be completely emotionally intimate with others, but I often find that others are reluctant to get as close as I would like. I am uncomfortable being without close relationships, but I sometimes worry that others don’t value me as much as I value them*); and Style D (*I am comfortable without close emotional relationships. It is very important to me to feel independent and self-sufficient, and I prefer not to depend on others or have others depend on me*).

*2.2.9.11 Modified Medical Outcome Social Support Survey* (mMOS-SSS) (Moser, Stuck, Silliman, Ganz, & Clough-Gorr, 2012). The 8-item mMOS-SSS, an abbreviated version of the 19-item MOS-SSS (Sherbourne & Stewart, 1991), ask respondents to report how frequently they have available to them two domains of social support (instrumental/tangible and emotional). Items include, “How often are each of the following kinds of support available to you if you need it: to help you if you were confined to bed?” and “to love you and make you feel wanted?”), and are scored on a 5-point Likert scale ranging from 1, none of the time to 5, all of the time. The measure had good internal reliability (Cronbach’s alpha range .88-.93) when used in studies of older women diagnosed with breast cancer (Moser et al., 2012).

**2.2.10 Health-related behaviours.**

*2.2.10.1 Body mass index.* Respondents were asked to report their best estimate of their height and weight and were able to do so using the imperial or metric system. They were asked to estimate their weight according to one of four categories: obese, overweight, normal weight, or underweight.

*2.2.10.2 Alcohol Use.* Respondents were asked to complete an adapted version of the 3-item AUDIT-C, which has good specificity and sensitivity for detecting alcohol dependence and at-risk drinking in the general population (Dawson, Grant, Stinson, & Zhou, 2005). The AUDIT-C questions were asked ‘During lockdown (in the last four months): (1) how often did you have a drink containing alcohol (response categories 1 ‘never’ to 5 ‘4 or more times a week’); (2) how many drinks containing alcohol did you have on a typical day when you were drinking (response categories 1 ‘1-2 drinks’ to 5 ’10 or more’; and (3) how often did you have six or more alcohol drinks on one occasion (response categories 1 ‘never’ to 5 ‘almost daily’. Respondents were also asked the extent to which they drank alcohol in the past week in the following settings: (1) on your own, in your house/garden; (2) with someone else in your own house/garden; (3) with family/friends online (e.g. WhatsApp, group Zoom/Skype); (4) in public (i.e. outside your house/garden) and (5) in a pub or restaurant. Responses were rated on a binary (yes/no) scale).

*2.2.10.3 Daily activities.* *Phase 1 only.* Respondents were asked about their engagement in a list of seven daily activities in the past week and asked to indicate, on how many days (response scale 1 ‘Not at all’ to 5 ‘Every day’), they had: (1) left the house for food, health reasons or work; (2) exercised outside the house once a day; (3) exercised outside the house more than once a day; (4) met up with friends or extended family outside home; (5) gathered in a group of more than 2 people in a park or public space; (6) driven to a national park or other green space to exercise; and (7) left the house to provide assistance to a vulnerable or elderly person.

**2.2.11 Authoritarianism and Conformity**

*2.2.11.1 Authoritarian Child Rearing Values (ACRV-2) Scale Child (American National Election Studies, 2014).* The forced choice child-rearing items were first introduced in the 1992 American National Election Study (ANES) as a brief measure of authoritarianism. Respondents were asked to consider the following statement: “'*Although there are a number of qualities that people feel that children should have, every person thinks that some are more important than others*.” Respondents were then presented with a pair of desirable qualities and asked to consider which one is more important for a child to have: (1) independence or respect for elders; (2) curiosity or good manners; (3) obedience or self-reliance; and (4) being considerate or well-behaved. Perez and Hetherington (2014) argue that the child-rearing items have good measurement properties (especially among White respondents); for example, in the 2008 ANES, a confirmatory factor analysis of the 4-item scale resulted in fit indices like the CFI and TLI above .90 and RMSEA well below .10. However, they find that the scale may operate differently for Black respondents at least in the US, and other scholars have confirmed these racial differences. Similarly, Hooper (2017) confirms that the ANES child-rearing items are a reliable measure and argues that the lower Cronbach's alpha for the scale, which has been reported to be in the .60s in other research (e.g., see Bizumic and Duckitt (2018)), underestimates its reliability (as does confirmatory factor analysis) because of the skewed marginal distributions induced by the forced choice response options. Adjusting the Cronbach's alpha on data from the 2016 ANES, for instance, results in a scale alpha of .81. More recently, Hooper (2020) argues that the items are probably better characterized as a measure of autonomy - conformity than they are as a pure measure of authoritarianism.

**2.2.12 Discrimination**

*2.2.12.1 Everyday Discrimination Scale Short Form* (EDS-SF) (Sternthal, Slopen, & Williams, 2011). *Phase 1 only.* A modified version of the 4-item EDS-SF was used to measure respondents’ perceived discrimination in terms of (1) being treated with less courtesy or respect compared to others, (2) having been threatened or harassed; (3) other people acting as if they were afraid of you; and (4) other people acting as if they think you are not smart, both before and during the pandemic. A 6-point Likert scale was presented for each act of perceived discrimination ranging from 1, ‘almost every day’ to 6’never’. Respondents who indicated that they had been discriminated against were asked to report the reasons why they believed they had been discriminated against in each situation from a list of 11 (e.g. gender, ethnicity, age, religion, height, sexual orientation, etc.).

**2.2.12 Political Behaviour and Attitudes**

*2.2.12.1 Voting behaviour and political party affiliation*. ***Phase 2*** respondents were asked to report their voting behaviour in response to the European Referendum (May 2016) (options voted Leave, voted Remain, did not vote, ineligible to vote). All respondents were asked to indicate: (1) the political party to which they most identify; (2) the party to whom they gave their 1^st^ preference vote in the 2019 general election; and (3) the party for whom they would be most likely to vote for if government elections were held today, with responses recorded as follows: (1) I was/am not eligible to vote; (2) I was eligible to vote but I did not vote/I would not vote, (3) Alliance Party of Northern Ireland; (4) BREXIT Party; (5) British National Party (BNP) (6) Change UK; (7) Conservative; (8) Democratic Unionist Party (DUP); (9) Green Party; (10) Labour; (11) Liberal Democrat; (12) Plaid Cymru; (13) Scottish National Party (SNP); (14) Sinn Féin; (15) Social Democratic and Labour Party (SDLP); (16) UK Independence Party (UKIP); (17) Ulster Unionist Party; or (18) Other (specify).

Adapting a question from the British Election Study 2015 (Fieldhouse et al., 2016), respondent were asked to indicate, with reference to the political party with which they most identify: “When I speak about this party, I usually say ‘we’ instead of ‘they’” and “When people criticize this party, it feels like a personal insult” – responses to both questions were scores on a 5-point Likert scale ranging from (1) Strongly disagree to (5) Strongly agree.

*Phase 2 only.* One additional question, adapted from the British Election Study 2017 Fieldhouse et al. (2018), asked respondents how they would describe their (1) political affiliation (on a 10-point scale ranging from 1 ‘left-wing’ to 10 ‘right-wing’).

*2.2.12.2 Trust in establishments.* Respondents were asked the extent to which they have trust in the following institutions/groups: (1) parliament; (2) the government; (3) the police; (4) the legal system; (5) political parties; (6) scientists; (7) doctors and other health professionals and (8) pharmaceutical companies. Responses were scored on a 5-point Likert scale ranging from 1 ‘completely trust’ to 5 ‘do not trust at all’.

**2.2.13 Miscellaneous. UK context-specific content – Summer 2020**

In light of on-going restrictions on domestic and international travel, the survey completed with a final question to ask respondents “*Will you be holidaying this summer*?” with the response categories: yes, abroad; yes, in the UK; maybe abroad; maybe in the UK; or no.

**References**

Allison, C., Auyeung, B., & Baron-Cohen, S. (2012). Toward brief “red flags” for autism screening: the short autism spectrum quotient and the short quantitative checklist in 1,000 cases and 3,000 controls. *Journal of the American Academy of Child & Adolescent Psychiatry, 51*(2), 202-212. e207.

American National Election Studies. (2014). User’s guide and codebook for the ANES 2012 time series study. In: University of Michigan and Stanford University MI.

American Psychiatric Association. (2000). *Diagnostic criteria from dsM-iV-tr*: American Psychiatric Pub.

Baron-Cohen, S., Scott, F. J., Allison, C., Williams, J., Bolton, P., Matthews, F. E., & Brayne, C. (2009). Prevalence of autism-spectrum conditions: UK school-based population study. *The British Journal of Psychiatry, 194*(6), 500-509.

Bartholomew, K., & Horowitz, L. M. (1991). Attachment styles among young adults: a test of a four-category model. *Journal of Personality and Social Psychology, 61*(2), 226.

Beck, A. T., Weissman, A., Lester, D., & Trexler, L. (1974). The measurement of pessimism: the hopelessness scale. *Journal of consulting and clinical psychology, 42*(6), 861.

Ben‐Ezra, M., Karatzias, T., Hyland, P., Brewin, C. R., Cloitre, M., Bisson, J. I., . . . Shevlin, M. (2018). Posttraumatic stress disorder (PTSD) and complex PTSD (CPTSD) as per ICD‐11 proposals: A population study in Israel. *Depression and Anxiety, 35*(3), 264-274.

Bizumic, B., & Duckitt, J. (2018). Investigating right wing authoritarianism with a very short authoritarianism scale. *Journal of Social and Political Psychology, 6*(1), 129-150. doi:10.5964/jspp.v6i1.835

Cloitre, M., Hyland, P., Bisson, J. I., Brewin, C. R., Roberts, N., Karatzias, T., & Shevlin, M. (2019). ICD-11 PTSD and complex PTSD in the United States: a population-based study. *Journal of Traumatic Stress, 32*(6), 833-842. doi:10.1002/jts.22454

Cloitre, M., Shevlin, M., Brewin, C. R., Bisson, J. I., Roberts, N. P., Maercker, A., . . . Hyland, P. (2018). The International Trauma Questionnaire: development of a self‐report measure of ICD‐11 PTSD and complex PTSD. *Acta Psychiatrica Scandinavica, 138*(6), 536-546.

Connor, K. M., Kobak, K. A., Churchill, L. E., Katzelnick, D., & Davidson, J. R. (2001). Mini‐SPIN: A brief screening assessment for generalized social anxiety disorder. *Depression and Anxiety, 14*(2), 137-140.

Dawson, D. A., Grant, B. F., Stinson, F. S., & Zhou, Y. (2005). Effectiveness of the derived Alcohol Use Disorders Identification Test (AUDIT‐C) in screening for alcohol use disorders and risk drinking in the US general population. *Alcoholism: Clinical and Experimental Research, 29*(5), 844-854.

de Vroege, L., Hoedeman, R., Nuyen, J., Sijtsma, K., & van der Feltz-Cornelis, C. M. (2012). Validation of the PHQ-15 for somatoform disorder in the occupational health care setting. *Journal of Occupational Rehabilitation, 22*(1), 51-58.

Department for Transport. (2020). Coronavirus (COVID-19): travel corridors List of countries, territories and regions from where you can travel to England and may not have to self-isolate. Retrieved from <https://www.gov.uk/guidance/coronavirus-covid-19-travel-corridors>

Elahi, A., Algorta, G. P., Varese, F., McIntyre, J., & Bentall, R. (2017). Do paranoid delusions exist on a continuum with subclinical paranoia? A multi-method taxometric study. *Schizophrenia Research, 190*, 77-81.

Everson, S. A., Goldberg, D. E., Kaplan, G. A., Cohen, R. D., Pukkala, E., Tuomilehto, J., & Salonen, J. T. (1996). Hopelessness and risk of mortality and incidence of myocardial infarction and cancer. *Psychosomatic Medicine, 58*(2), 113-121.

Fieldhouse, E., Green, J., Evans, G., Schmitt, H., van der Eijk, C., Mellon, J., & Prosser, C. (2016). British Election Study, 2015: Face-to-Face Post-Election Survey.

Fieldhouse, E., Green, J., Evans, G., Schmitt, H., van der Eijk, C., Mellon, J., & Prosser, C. (2018). *British Election Study, 2017: Face-to-Face Post-Election Survey* Retrieved from <https://www.britishelectionstudy.com/wp-content/uploads/2019/01/BES-2017-F2F-codebook.pdf>

Fraser, L., Burnell, M., Salter, L. C., Fourkala, E.-O., Kalsi, J., Ryan, A., . . . Menon, U. (2014). Identifying hopelessness in population research: a validation study of two brief measures of hopelessness. *BMJ Open, 4*(5).

Health and Social Care Public Health Agency. (2020). StopCOVID NI contact tracing app. Retrieved from <https://www.publichealth.hscni.net/covid-19-coronavirus/testing-and-tracing-covid-19/stopcovid-ni-contact-tracing-app>

Hooper, M. J. (2017). The NES child rearing scale: A detailed empirical examination of its measurement properties. *Available at SSRN 3050038*. doi:10.2139/ssrn.3050038

Hooper, M. J. (2020). The ANES Child Rearing Scale: Is It a Measure of Authoritarianism? *Available at SSRN 3597712*. doi:10.2139/ssrn.3597712

Hughes, M. E., Waite, L. J., Hawkley, L. C., & Cacioppo, J. T. (2004). A short scale for measuring loneliness in large surveys: Results from two population-based studies. *Research on Aging, 26*(6), 655-672.

Hyland, P., Shevlin, M., Brewin, C. R., Cloitre, M., Downes, A., Jumbe, S., . . . Roberts, N. (2017). Validation of post‐traumatic stress disorder (PTSD) and complex PTSD using the International Trauma Questionnaire. *Acta Psychiatrica Scandinavica, 136*(3), 313-322.

Karatzias, T., Shevlin, M., Fyvie, C., Hyland, P., Efthymiadou, E., Wilson, D., . . . Cloitre, M. (2016). An initial psychometric assessment of an ICD-11 based measure of PTSD and complex PTSD (ICD-TQ): Evidence of construct validity. *Journal of Anxiety Disorders, 44*, 73-79.

Kroenke, K., Spitzer, R. L., & Williams, J. B. (2001). The PHQ‐9: validity of a brief depression severity measure. *Journal of General Internal Medicine, 16*(9), 606-613.

Kroenke, K., Spitzer, R. L., & Williams, J. B. (2002). The PHQ-15: validity of a new measure for evaluating the severity of somatic symptoms. *Psychosomatic Medicine, 64*(2), 258-266.

Kroenke, K., Spitzer, R. L., Williams, J. B., & Löwe, B. (2010). The patient health questionnaire somatic, anxiety, and depressive symptom scales: a systematic review. *General Hospital Psychiatry, 32*(4), 345-359.

Kyriazos, T. A., Stalikas, A., Prassa, K., Galanakis, M., Yotsidi, V., & Lakioti, A. (2018). Psychometric Evidence of the Brief Resilience Scale (BRS) and Modeling Distinctiveness of Resilience from Depression and Stress. *Psychology, 9*(7), 1828-1857.

Levenson, H. (1973). Multidimensional locus of control in psychiatric patients. *Journal of Consulting and Clinical Psychology, 41*(3), 397.

Löwe, B., Decker, O., Müller, S., Brähler, E., Schellberg, D., Herzog, W., & Herzberg, P. Y. (2008). Validation and standardization of the Generalized Anxiety Disorder Screener (GAD-7) in the general population. *Medical Care*, 266-274.

Lyubomirsky, S., & Lepper, H. S. (1999). A measure of subjective happiness: Preliminary reliability and construct validation. *Social Indicators Research, 46*(2), 137-155.

McIntyre, J. C., Wickham, S., Barr, B., & Bentall, R. P. (2018). Social identity and psychosis: Associations and psychological mechanisms. *Schizophrenia Bulletin, 44*(3), 681-690.

McManus, S., Bebbington, P., Jenkins, R., & Brugha, T. (2016). Mental health and wellbeing in England: Adult psychiatric morbidity survey 2014. Retrieved from <https://digital.nhs.uk/data-and-information/publications/statistical/adult-psychiatric-morbidity-survey/adult-psychiatric-morbidity-survey-survey-of-mental-health-and-wellbeing-england-2014>

Melo, S., Corcoran, R., Shryane, N., & Bentall, R. P. (2009). The persecution and deservedness scale. *Psychology and Psychotherapy: Theory, Research and Practice, 82*(3), 247-260.

Moser, A., Stuck, A. E., Silliman, R. A., Ganz, P. A., & Clough-Gorr, K. M. (2012). The eight-item modified Medical Outcomes Study Social Support Survey: psychometric evaluation showed excellent performance. *Journal of Clinical Epidemiology, 65*(10), 1107-1116.

NHS Scotland Test and Protect. (2020). Protect Scotland. Retrieved from <https://protect.scot/>

NHS Test and Trace. (2020). Download the NHS COVID-19 app today. Retrieved from <https://www.covid19.nhs.uk/>

Perez, E. O., & Hetherington, M. J. (2014). Authoritarianism in black and white: Testing the cross-racial validity of the child rearing scale. *Political Analysis*, 398-412.

Radloff, L. S. (1977). The CES-D scale: A self-report depression scale for research in the general population. *Applied Psychological Measurement, 1*(3), 385-401.

Rammstedt, B., & John, O. P. (2007). Measuring personality in one minute or less: A 10-item short version of the Big Five Inventory in English and German. *Journal of Research in Personality, 41*(1), 203-212.

Robins, R. W., Hendin, H. M., & Trzesniewski, K. H. (2001). Measuring global self-esteem: Construct validation of a single-item measure and the Rosenberg Self-Esteem Scale. *Personality and Social Psychology Bulletin, 27*(2), 151-161.

Rodríguez-Rey, R., Alonso-Tapia, J., & Hernansaiz-Garrido, H. (2016). Reliability and validity of the Brief Resilience Scale (BRS) Spanish Version. *Psychological Assessment, 28*(5), e101.

Sapp, S. G., & Harrod, W. J. (1993). Reliability and validity of a brief version of Levenson's locus of control scale. *Psychological Reports, 72*(2), 539-550.

Sherbourne, C. D., & Stewart, A. L. (1991). The MOS social support survey. *Social science & medicine, 32*(6), 705-714.

Smith, B. W., Dalen, J., Wiggins, K., Tooley, E., Christopher, P., & Bernard, J. (2008). The brief resilience scale: assessing the ability to bounce back. *International Journal of Behavioral Medicine, 15*(3), 194-200.

Spitzer, R. L., Kroenke, K., Williams, J. B., & Löwe, B. (2006). A brief measure for assessing generalized anxiety disorder: the GAD-7. *Archives of Internal Medicine, 166*(10), 1092-1097.

Sternthal, M. J., Slopen, N., & Williams, D. R. (2011). Racial disparities in health: How much does stress really matter? *Du Bois review, 8*(1), 95-113.

Tomás-Sábado, J., Gómez-Benito, J., & Limonero, J. T. (2005). The death anxiety inventory: A revision. *Psychological Reports, 97*(3), 793-796.

UK Cabinet Office/Department of Education. (2020). Guidance: Critical workers who can access schools or educational settings. Retrieved from <https://www.gov.uk/government/publications/coronavirus-covid-19-maintaining-educational-provision/guidance-for-schools-colleges-and-local-authorities-on-maintaining-educational-provision>

Vallières, F., Ceannt, R., Daccache, F., Abou Daher, R., Sleiman, J., Gilmore, B., . . . Hyland, P. (2018). ICD‐11 PTSD and complex PTSD amongst Syrian refugees in Lebanon: the factor structure and the clinical utility of the International Trauma Questionnaire. *Acta Psychiatrica Scandinavica, 138*(6), 547-557.
