# Supplementary material for: Context, design and conduct of the longitudinal COVID‐19 psychological research consortium study–wave 3
Source: Int J Methods Psychiatr Res. 2021 May 22;30(3):e1880. doi: 10.1002/mpr.1880 (PMC8209941; doi:10.1002/mpr.1880)
Supplement: Supplementary file 2 — Supplementary Material [file MPR-30-e1880-s001.docx]

Supplementary Table 1 Description of the Socio-political Context^1^ of COVID-19 in the UK, including Number of Confirmed Cases of COVID-19 and confirmed Deaths due to COVID-19^2^ in the UK (May-August 2020)

| **Date** | **Key event** | **Number of confirmed** | |
| --- | --- | --- | --- |
|  |  | **Cases** | **Deaths** |
| 1-5-20 | SSHSC, Matt Hancock, confirms the government's target of providing (but not necessarily completing) 100,000 tests a day by the end of April has been met. | 177,454 | 27,510 |
| 5-5-20 | The UK records the highest number of COVID-19 related deaths in Europe. Trials of the NHS contact-tracing app start on the Isle of Wight. | 190,584 | 28,734 |
| 7-5-20 | The Bank of England predicts that the economy will shrink 14% in 2020 due to the impact of COVID-19. Baroness Dido Harding is appointed to lead the government's programme of testing and tracing. | 201,101 | 30,076 |
| 10-5-20 | In a televised address, PM, Boris Johnson, outlines changes to lockdown restrictions in England and updates the government’s coronavirus message from "stay at home, protect the NHS, save lives" to "**stay alert**, control the virus, save lives". The original ‘stay at home’ slogan remains in place in Scotland, Wales, and Northern Ireland. A new COVID-19 Alert Level system is announced, ranging from green (level one) to red (level five), similar to the UK Terror Threat Levels. PM announces that passengers arriving in the UK on international flights will soon be asked to go into quarantine for 14 days. |  |  |
| 11-5-20 | The UK government advises people in England to wear face coverings in enclosed spaces where social distancing is not possible, such as on public transport and in shops. | 223,060 | 32,065 |
| 12-5-50 | Chancellor, Rishi Sunak, extends the UK's furlough scheme until October 2020, with employees continuing to receive 80% of their monthly wages up to £2,500. A quarter of the UK workforce, some 7.5 million people, are now covered by the scheme, costing £14bn a month. | 226,463 | 32,692 |
| 13-5-20 | The Health Protection (Coronavirus, Restrictions) (England) (Amendment No. 2) Regulations 2020 (SI 500) come into effect, allowing the re-opening of garden centres, sports courts, and recycling centres. In addition to outdoor exercise, open-air recreation is also permitted with no more than one member of another household. The UK economy reported shrank by 2% in the first quarter of 2020 and the Chancellor announces that the country is “*very likely*” in “*significant recession*”. |  |  |
| 15-5-20 | UK government scientists and teaching unions hold talks in a bid to safely reopen schools. | 233,151 | 33,614 |
| 17-5-20 | A further £84m of funding is announced to help mass-produce a COVID-19 vaccine being trialled by the University of Oxford and that an agreement has been secured with pharmaceutical company AstraZeneca to manufacture the vaccine, and distributed it to the UK first. |  |  |
| 18-5-20 | The four UK Chief Medical Officers add loss of smell or taste is added to the list of coronavirus symptoms. SSHSC, Matt Hancock, announces that anyone in the UK over the age of five with symptoms can now be tested for COVID-19. It is also confirmed that 21,000 contact tracers have been recruited across the UK and are ready to begin work. At the daily briefing in Downing Street, FFS, Dominic Rabb, states that it is “*not sustainable*” to keep the lockdown in place “*permanently*”. | 243,695 | 34,636 |
| 19-5-20 | Data from the ONS indicate that the number of people claiming Jobseeker’s Allowance increased by 856,000 in April, to 2.1 million. Major security issues are identified during the pilot of the NHS COVID-19 tracing app and there are calls for new legislation to prevent officials using the data collected for purposes other than identifying those at risk from the virus. | 246,406 | 34,796 |
| 20-5-20 | PM, Boris Johnson, confirms that a track and trace system will be in place from 1 June. The UK government faces mounting pressure from councils and teaching unions to reconsider its plans to reopen primary schools from 1 June. The number of people in hospital with COVID-19 drops below 10,000 for the first time since March 2020. | 248,818 | 35,341 |
| 22-5-20 | HS, Priti Patel, announces new quarantine rules for all travellers arriving in the UK (except for some limited circumstances), requiring them to self-isolate for 14 days from 8 June, to help protect against a second wave of coronavirus. ONS reports that government borrowing rose to £62bn in April, the highest monthly figure on record. | 254,195 | 36,393 |
| 23-5-20 | Dominic Cummings, the PM’s chief political adviser, comes under mounting pressure to resign after media reports emerge claiming that he travelled 260 miles from London to Durham to self-isolate during lockdown and while his wife was displaying COVID-19 symptoms. |  |  |
| 24-5-20 | PM confirms plans for the phased reopening of schools in England from 1 June for younger children (below Year 6) and from 15 June for some older year groups, to facilitate examination preparations. |  |  |
| 25-5-20 | Dominic Cummings gives a detailed explanation of his actions during lockdown at a press conference in the Downing Street Rose Garden. | 261,184 | 36,914 |
| 26-5-20 | Death registration figures for the week ending 15 May show the lowest number of COVID-19 deaths since the beginning of April. | 265,227 | 37,048 |
| 27-5-20 | Figures from HM Treasury indicate the Coronavirus Job Retention Scheme for furloughed workers in the UK now covers 8.4 million people, an increase of 400,000 from the previous week. |  |  |
| 28-5-20 | Contact tracing systems go live in England and Scotland. |  |  |
| 29-5-20 | Chancellor, Rishi Sunak, announces that the Coronavirus Job Retention Scheme will end at the end of October 2020. Self-employed people whose work has been affected by the outbreak will receive a "second and final" government grant in August 2020. |  |  |
| 1-6-20 | The Health Protection (Coronavirus, Restrictions) (England) (Amendment No. 3) Regulations 2020 (SI 558) come into effect, signalling the re-opening of car and caravan showrooms, outdoor sports amenities, and outdoor non-food markets. Prohibitions on leaving home are replaced by a prohibition on staying overnight away from home, with certain specific exceptions. Gatherings of people from more than one household are limited to six people outdoors and are prohibited entirely indoors, with exceptions including education. | 274,762 | 38,489 |
| 3-6-20 | HS, Priti Patel, confirms plans to introduce a 14-day quarantine for new arrivals into the UK. Those who break the quarantine conditions in England will face fines of £1,000 and potential prosecution. | 279,856 | 39,728 |
| 5-6-20 | The number of recorded deaths in the UK passes 40,000. |  |  |
| 6-6-20 | Anti-racism demonstrations are held in cities across the UK; attendees are reported to be in the thousands. |  |  |
| 7-6-20 | Thousands of protesters take part in a second day of anti-racism demonstrations in cities across the UK. |  |  |
| 8-6-20 | Rules requiring travellers arriving in the UK to quarantine for 14 days come into force. The UK COVID-19 daily death record is 55, the lowest since lockdown was introduced on 23 March. | 286,194 | 40,542 |
| 9-6-20 | It is confirmed that all non-essential retailers in England can reopen from Monday 15 June providing they follow safety guidelines. A quarter of the UK workforce (8.9 million workers) are now covered by the Coronavirus Job Retention Scheme at a cost of £19.6bn to date. Thirty medical organisations representing black, Asian and minority ethnic doctors and nurses write to SSHSC, Matt Hancock, and EM, Kemi Badenoch, expressing their concern at a government review that found black, Asian and ethnic minority people are twice as likely to die from COVID-19. | 289,140 | 40,883 |
| 11-6-20 | SSHSC, Matt Hancock, describes participation with the Test and Trace programme as a "*civic duty*". | 291,409 | 41,279 |
| 12-6-20 | ONS data confirm that the UK economy shrunk by 20.4% in April, the largest monthly contraction on record. | 292,950 | 41,481 |
| 13-6-20 | Parts of the Health Protection (Coronavirus, Restrictions) (England) (Amendment No. 4) Regulations 2020 (SI 588) come into effect. These largely relate to the introduction of “support bubbles” – that households with one adult may now become linked with one other household of any size for the purposes of permitted gatherings and overnight stays. The rules on gatherings are also relaxed to allow medical appointments and births to be accompanied, and to permit some visits to people in hospital, hospices, and care homes. |  |  |
| 15-6-20 | The remainder of the Health Protection (Coronavirus, Restrictions) (England) (Amendment No. 4) Regulations 2020 (SI 588) comes into effect, allowing the general re-opening of some retail shops and public-facing businesses. Outdoor animal-related attractions such as farms, zoos and safari parks may also re-open. Places of worship may again be used for private prayer (but not for communal worship). Libraries in England remain closed. | 295,889 | 41,698 |
| 16-6-20 | ONS figures suggest more than 600,000 people have lost their jobs between March and May 2020 because of the COVID-19 outbreak. | 296,857 | 41,736 |
| 18-6-20 | The Bank of England announces plans to inject an extra £100bn into the UK economy to help fight downturn precipitated by the pandemic. A deal to begin the manufacture of a potential vaccine, even though it has yet to receive clinical approval, has been reached between AstraZeneca and the University of Oxford. The plan is to stockpile the vaccine ready for its approval. Those over the age of 50 and with certain underlying health conditions will then be prioritised for the vaccine once launched. | 299,251 | 42,153 |
| 19-6-20 | The UK's COVID-19 Alert Level is lowered from Level 4 (severe risk, high transmission) to Level 3 (substantial risk, general circulation). HM Treasury estimates that UK debt stands at £1.95trn and is larger than the economy for the first time in 50 years, following a record amount of borrowing in May. | 300,469 | 42,288 |
| 22-6-20 | The UK records its lowest number of new coronavirus cases since the beginning of lockdown; the number of deaths recorded for the previous day stands at 15, the lowest figure since 15 March. | 304,331 | 42,632 |
| 24-6-20 | Scientists at Imperial College London begin human trials of a COVID-19 vaccine after tests on animals indicate an effective immune response | 306,210 | 442,927 |
| 26-6-20 | The UK government confirms that people returning from certain countries (to be confirmed at a later date) will not be required to quarantine for 14 days when returning to the UK from 6 July. |  |  |
| 29-6-20 | SSHSC, Matt Hancock, announces a local lockdown for Leicester city, including the closure of non-essential retailers from 30 June, and the closure of schools from 2 July. People in Leicester are advised to stay at home as much as possible, while it is recommended that all but essential travel to, from and within the city should be avoided. | 311,965 | 43,575 |
| 30-6-20 | PM, Boris Johnson, sets out a £5bn post-coronavirus recovery plan for the UK that will see home building and improvements to infrastructure, describing it as a "*new deal*". |  |  |
| 1-7-20 | The UK government published a list of 59 ‘low risk’ countries for which quarantine will not apply when arriving back in England as from 10 July. They include Greece, France, Belgium, and Spain, but Portugal and the United States are among those not on the list. These changes do not apply to Scotland, Wales, or Northern Ireland, where quarantine restrictions remain in place for all arrivals from outside the UK. | 312,654 | 43,730 |
| 4-7-20 | The Health Protection (Coronavirus, Restrictions) (No. 2) (England) Regulations 2020 came into force in England allowing pubs, restaurants, and hairdressers to reopen in England. Under these regulations, the Secretary of State also has powers to make declarations restricting access to public outdoor places. Leicester was excluded from the relaxations due to its high rate of COVID-19. |  |  |
| 6-7-20 | The UK government announces grants and loans of £1.57bn to support theatres, galleries, museums, and other cultural venues affected by the COVID-19 outbreak. | 285,416* | 44,220 |
| 8-7-20 | Chancellor, Rishi Sunak, unveils a £30bn spending package aimed at mitigating the economic impact of the COVID-19 pandemic, including a temporary reduction in VAT for the hospitality sector, a scheme to pay firms £1,000 for each employee brought back from furlough, a scheme to get young people into employment, and a temporary rise in the stamp duty threshold. | 286,349 | 44,391 |
| 9-7-20 | **Soft-launch (piloting) of the C19PRC-UKW3, followed by commencement of field work.** | 286,979 | 44,517 |
| 10-7-20 | Quarantine rules are relaxed for people arriving in the UK from 75 countries and overseas territories. | 287,621 | 44,602 |
| 11-7-20 | Parts of The Health Protection (Coronavirus, Restrictions) (No. 2) (England) (Amendment) Regulations 2020 come into effect, allowing outdoor swimming pools and water parks to re-open. |  |  |
| 13-7-20 | The remainder of The Health Protection (Coronavirus, Restrictions) (No. 2) (England) (Amendment) Regulations 2020 comes into effect, allowing the re-opening of nail bars and salons, tanning booths and salons, spas and beauty salons, massage parlours, tattoo parlours, and body and skin piercing services. | 289,603 | 44,819 |
| 15-7-20 | A temporary cut in VAT worth £4bn comes into force until 12 January 2021. | 291,373 | 44,968 |
| 16-7-20 | SSHSC, Matt Hancock, announces that the parts of the Leicester local lockdown are to be extended. | 291,911 | 45,053 |
| 17-7-20 | PM announces a further easing of lockdown restrictions for England, with plans for a "*significant return to normality*" by Christmas. Use of public transport for non-essential journeys is permitted with immediate effect. An extra £3bn for NHS in England is provided to help prepare for a possible second wave of COVID-19 over the coming winter. | 292,552 | 45,119 |
| 18-7-20 | Local authorities in England get new powers to close shops and outdoor public spaces, and to cancel events in order to control COVID-19. Public Health England pauses publication of daily death statistics to review the way these are reported. |  |  |
| 20-7-20 | The UK has signed a deal for 90 million doses of promising vaccines being developed jointly by the pharmaceutical companies  and Pfizer, and by Valneva. This is in addition to the 100 million doses being developed by the University of Oxford and AstraZeneca. | 294,792 | $ |
| 21-7-20 | Chancellor, Rishi Sunak, announces that 900,000 public sector workers will get an above-inflation pay rise of 3.1% in acknowledgement of the important role they have played during the pandemic. Health experts tell the House of Commons Health Committee that even with a vaccine the UK is likely to be living with COVID-19 for many years. | 295,372 |  |
| 23-7-20 | Northern Ireland's Department of Health confirms the release of Northern Ireland's contact-tracing app, StopCOVID NI, for as early as 29 July. Northern Ireland is the first part of the UK to launch a contact-tracing app. | 296,377 |  |
| 24-7-20 | Face coverings become compulsory in shops and most other enclosed public places in England. ONS figures show that retail sales increased almost to pre-lockdown levels in June. | 297,146 |  |
| 25-7-20 | Public Health England warns that being obese and overweight puts people at greater risk of severe illness or death as a result of COVID-19. |  |  |
| 26-7-20 | The UK changes its advice on travel to Spain, advising against all non-essential travel to mainland Spain, the Balearic, and Canary Islands. |  |  |
| 30-7-20 | An extension of the self-isolation period from 7 to 10 days for people who test positive for, or show signs of, the virus is announced. Local restrictions were imposed at midnight for Greater Manchester, and parts of East Lancashire and Yorkshire. Northern Ireland's contact tracing app StopCOVID NI is launched. | 301,455 |  |
| 31-7-20 | ONS household survey indicates COVID-19 cases in England are rising again. |  |  |
| 9-8-20 | **Fieldwork for C19PRC-UKW3 ends.** | 310,825 |  |

Note. PM=Prime Minister; SSHSC= Secretary of State for Health and Social Care; FFS=First Secretary of State and Secretary of State for Foreign and Commonwealth Affairs; Business Secretary= BS; Office for National Statistics= ONS; HM= Her Majesty’s; HS=Home Secretary; EM=Equalities Minister. $: Official reporting of daily COVID-19 death rate by Public Health England paused and reviewed for accuracy

Sources: 1 <https://seneddresearch.blog/2020/03/19/covid-19-timeline-welsh-and-uk-governments-response/>

<https://en.wikipedia.org/wiki/Timeline_of_the_COVID-19_pandemic_in_the_United_Kingdom>

2 <https://www.mims.co.uk/live-updates-coronavirus-covid-19-uk/infections-and-infestations/article/1673649>

$ <https://www.independent.co.uk/news/uk/politics/coronavirus-uk-death-toll-nhs-phe-covid-19-government-england-scotland-a9626336.html>

Supplementary Table 2 Regression estimates predicting respondent participation at C19PRC-W3 by baseline characteristics.

| Wave 1 (baseline) respondent characteristics (March 2020) | | Present at C19PRC-UKW3  Odds ratio (95% confidence interval) |
| --- | --- | --- |
| Gender | Male | 1 |
|  | Female | 0.99 (0.97-1.01) |
| Age group (years) | 18-24 years | 1 |
|  | 25-34 years | 2.12 (1.47-3.07)** |
|  | 35-44 years | 2.67 (1.82-3.92)** |
|  | 45-54 years | 4.41 (3.04-6.42)** |
|  | 55-64 years | 6.34 (4.22-9.53)** |
|  | 65+ years | 7.35 (4.64-11.65)** |
| 2019 household income | ≤£15.490 | 1 |
|  | £15,491-£25,340 | 1.27 (0.91-1.77) |
|  | £25,341-£38,740 | 1.17 (0.82-1.66) |
|  | £38,741-£57,903 | 1.18 (0.83-1.67) |
|  | ≥£57,931 | 1.43 (0.99-2.05) |
| Economic activity | Employed (full or part-time) | 1.05 (0.82-1.35) |
|  | Other | 1 |
| Ethnicity | White | 0.97 (0.68-1.39) |
|  | Other | 1 |
| Birthplace | Born in UK | 1.37 (0.93-2.01) |
|  | Born elsewhere | 1 |
| Place of residence | City | 0.85 (0.67-1.07) |
|  | Suburb/Town/Rural | 1 |
| Educational attainment | Post-secondary education | 1.22 (0.99-1.51) |
|  | Did not attend post-secondary education | 1 |
| Household characteristics | Single adult household | 1.05 (0.79-1.40) |
|  | Other | 1 |
|  | Children under 18 years living in household | 0.62 (0.49-0.79)** |
|  | Other | 1 |
| Physical health | Chronic health condition | 0.79 (0.60-1.03) |
|  | None | 1 |
| Mental-health and psychological characteristics | Currently receiving/history of receiving treatment for mental health problems | 1.00 (0.80-1.27) |
|  | Other | 1 |
|  | Depression - PHQ-9 caseness met | 0.87 (0.62-1.21) |
|  | Not met | 1 |
|  | Anxiety - GAD-7 caseness met | 1.36 (0.97-1.88) |
|  | Not met | 1 |
|  | PTSD caseness met | 1.22 (0.90-1.67) |
|  | Not met | 1 |
|  | Loneliness caseness met | 0.97 (0.77-1.22) |
|  | Not met | 1 |
|  | Neuroticism | 0.95 (0.89-1.02) |
|  | Resilience | 0.99 (0.97-1.03) |
|  | Somatisation | 0.96 (0.94-0.98)* |
|  | Paranoia | 0.98 (0.96-1.01) |
|  | Death anxiety | 0.99 (0.98-1.00) |
|  | Intolerance of uncertainty | 1.03 (1.01-1.04)* |
|  | COVID-19 anxiety | 0.99 (0.99-1.01) |

**p*<0.01, ***p*<0.001

Supplementary Table 3 Outcome of raking weighting procedure

| Wave 1 variables | Wave 1 sample  (n=2025) | Wave 3 recontacts (Phase 1) (not weighted)  (n=1166) | W3 recontacts (weighted)  (n=1166) |  |
| --- | --- | --- | --- | --- |
| **Age** |  |  |  |  |
| 18-24 | 246 (12.1%) | 68 (5.8%) | 142 (12.1%) |  |
| 25-34 | 380 (18.8%) | 171 (14.7%) | 219 (18.8%) |  |
| 35-44 | 353 (17.4%) | 180 (15.4%) | 203 (17.4%) |  |
| 45-54 | 410 (20.2%) | 267 (22.9%) | 236 (20.2%) |  |
| 55-64 | 349 (17.2%) | 256 (22.0%) | 201 (17.2%) |  |
| 65+ | 287 (14.2%) | 224 (19.2%) | 165 (14.2%) |  |
| **Gender*** |  |  |  |  |
| Male | 972 (48.0%) | 620 (53.2%) | 560 (48.0%) |  |
| Female | 1053 (52.0%) | 546 (46.8%) | 606 (52.0%) |  |
|  |  |  |  |  |
| **Income (per annum)** | |  |  |  |
| ≤£15.490 | 410 (20.2%) | 224 (19.2%) | 236 (20.2%) |  |
| £15,491-£25,340 | 410 (20.2%) | 216 (18.5%) | 236 (20.2%) |  |
| £25,341-£38,740 | 385 (19.0%) | 212 (18.2%) | 222 (19.0%) |  |
| £38,741-£57,903 | 410 (20.2%) | 245 (21.0%) | 236 (20.2%) |  |
| ≥£57,931 | 410 (20.2%) | 269 (23.1%) | 236 (20.2%) |  |
| **Urbanicity** |  |  |  |  |
| City | 498 (24.6%) | 243 (20.8%) | 287 (24.6%) |  |
| Suburb/Town/Rural | 1527 (75.4%) | 923 (79.2%) | 879 (75.4%) |  |
| **Ethnicity** |  |  |  |  |
| White | 1848 (91.2%) | 1088 (93.4%) | 1067 (91.5%) |  |
| Non-white | 177 (8.7%) | 78 (6.7%) | 99 (8.5%) |  |
| **Household composition** |  |  |  |  |
| Children in household | 592 (29.2%) | 263 (22.6%) | 341 (29.2%) |  |
| No children in household | 1433 (70.8%) | 903 (77.4%) | 825 (70.8%) |  |
| **Born or raised in UK** |  |  |  |  |
| Yes | 1891 (93.4%) | 1101 (94.4%) | 1101 (94.4%) |  |
| No | 134 (6.6%) | 65 (5.6%) | 65 (5.6%) |  |
| **Mental disorders** |  |  |  |  |
| Depression - PHQ-9 caseness met | 448 (22.1%) | 206 (17.7%) | 244 (20.9%) |  |
| Anxiety - GAD-7 caseness met | 438 (21.6%) | 215 (18.4%) | 248 (21.2%) |  |
| PTSD caseness met | 340 (16.8%) | 158 (13.6%) | 196 (16.8%) |  |

*Other gender categories combined with ‘female’ for the purposes of creating raking weights.
